# Analysis of error profiles in deep next-generation sequencing data

**DOI:** 10.1186/s13059-019-1659-6

**Published:** 2019-03-14

**Authors:** Xiaotu Ma, Ying Shao, Liqing Tian, Diane A. Flasch, Heather L. Mulder, Michael N. Edmonson, Yu Liu, Xiang Chen, Scott Newman, Joy Nakitandwe, Yongjin Li, Benshang Li, Shuhong Shen, Zhaoming Wang, Sheila Shurtleff, Leslie L. Robison, Shawn Levy, John Easton, Jinghui Zhang

**Affiliations:** 1 Department of Computational Biology, St. Jude Children’s Research Hospital, Memphis, TN 38105 USA; 2 Department of Pathology, St. Jude Children’s Research Hospital, Memphis, TN 38105 USA; 3 Key Laboratory of Pediatric Hematology and Oncology Ministry of Health, Department of Hematology and Oncology, Shanghai Children’s Medical Center, Shanghai Jiao Tong University School of Medicine, Shanghai, 200127 China; 4 Department of Epidemiology and Cancer Control, St. Jude Children’s Research Hospital, Memphis, TN 38105 USA; 5 HudsonAlpha Institute for Biotechnology, Huntsville, AL 35806 USA

**Keywords:** Deep sequencing, Error rate, Substitution, Subclonal, Detection, Hotspot mutation

## Abstract

**Background:**

Sequencing errors are key confounding factors for detecting low-frequency genetic variants that are important for cancer molecular diagnosis, treatment, and surveillance using deep next-generation sequencing (NGS). However, there is a lack of comprehensive understanding of errors introduced at various steps of a conventional NGS workflow, such as sample handling, library preparation, PCR enrichment, and sequencing. In this study, we use current NGS technology to systematically investigate these questions.

**Results:**

By evaluating read-specific error distributions, we discover that the substitution error rate can be computationally suppressed to 10^−5^ to 10^−4^, which is 10- to 100-fold lower than generally considered achievable (10^−3^) in the current literature. We then quantify substitution errors attributable to sample handling, library preparation, enrichment PCR, and sequencing by using multiple deep sequencing datasets. We find that error rates differ by nucleotide substitution types, ranging from 10^−5^ for A>C/T>G, C>A/G>T, and C>G/G>C changes to 10^−4^ for A>G/T>C changes. Furthermore, C>T/G>A errors exhibit strong sequence context dependency, sample-specific effects dominate elevated C>A/G>T errors, and target-enrichment PCR led to ~ 6-fold increase of overall error rate. We also find that more than 70% of hotspot variants can be detected at 0.1 ~ 0.01% frequency with the current NGS technology by applying in silico error suppression.

**Conclusions:**

We present the first comprehensive analysis of sequencing error sources in conventional NGS workflows. The error profiles revealed by our study highlight new directions for further improving NGS analysis accuracy both experimentally and computationally, ultimately enhancing the precision of deep sequencing.

**Electronic supplementary material:**

The online version of this article (10.1186/s13059-019-1659-6) contains supplementary material, which is available to authorized users.

## Background

Detecting somatic mutations present at a low frequency through deep sequencing is important for cancer genomic profiling [1]. Typical applications include detecting subclonal pathogenic mutations in driver genes such as *NRAS*/*KRAS* in leukemias that frequently seed relapse [2], mosaic cancer predisposition mutations [3, 4], age-related clonal hematopoiesis [5] that increases cancer risk, and liquid biopsy for non-invasive diagnosis and disease monitoring [6–9].

Errors acquired during next-generation sequencing (NGS) are key confounding factors of sensitive detection of low-frequency variants by deep sequencing. The substitution error rate by conventional NGS was first reported to be > 0.1% in 2011 [10] and was similar in later reports [11, 12] and in a recent review [1]. This presumed high error rate (> 0.1%) constrains further exploration of ways to improve sensitivity of low-frequency variant detection. For example, the FDA-authorized MSKCC-IMPACT study reported a detection limit of 0.02 mutant allele fraction (MAF) for hotspot mutations and 0.05 for non-hotspot mutations at a read-depth of 500–1000X [13]. With the rapid progress in sequencing technology and dramatic reductions in sequencing cost, there is a great need to systematically evaluate sequencing errors at various steps of a conventional NGS workflow, as this knowledge will help improve low-level variant detection by deep sequencing.

In this study, we performed a comprehensive analysis of the substitution errors in deep sequencing data using the conventional NGS technology. We focused on substitution variants because they are the most abundant mutation type in both adult (97%) [14] and pediatric cancers (93%) [15, 16]. We first explored error profiles by performing a paired cancer-normal dilution experiment followed by deep sequencing and discovered that the substitution error rate can be suppressed computationally to 10^−5^ to 10^−4^, which is 10- to 100-fold lower than the current reports. We next analyzed distinct error profiles that can be attributed to different steps of NGS workflows, including sample handling, polymerase errors, and PCR enrichment steps. These results provide important insights for future improvements of sequencing accuracy.

## Results

### Study design

**Fig. 1:**
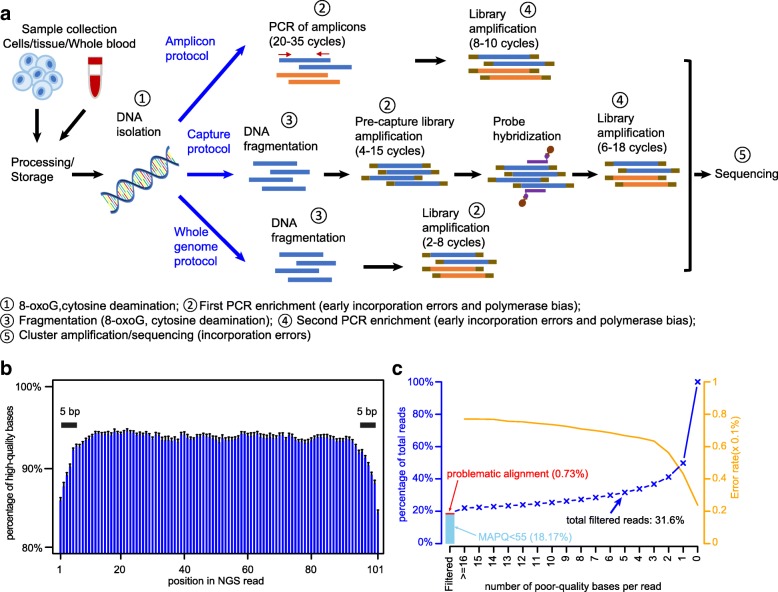
Potential error sources in next-generation sequencing workflow. **a** Illustration of the major steps of a typical next-generation sequencing workflow. Targeted deep sequencing is usually done by amplicon protocol or hybridization-capture protocol. Potential error sources are indicated by numbers. **b** Percentage of high-quality (Q30) bases by position in NGS read. This shows that the first and the last 5 bp have lower percentages of high-quality bases than do other positions. **c** Cumulative plot of NGS read quality distribution categorized by low-quality mapping (MAPQ < 55), potentially problematic alignment (“Methods”), and number of poor-quality bases in read (from ≥ 16 bp to 0 bp per read)

A typical NGS workflow involves multiple steps (Fig. 1a) prior to sequencing, including sample processing, DNA isolation, and PCR amplification. Errors can be introduced in each of these steps. For example, C>A/G>T errors have been reported to be due to DNA damage during sample processing [17, 18]. Spontaneous deamination of methylated cytosine to uracil [1, 19] can cause C>T/G>A errors. Additional errors can also be introduced by target-enrichment PCR and the sequencing step [1].

In this study, we systematically investigated substitution error profiles by analyzing multiple sequencing datasets from five DNA sequencing providers: three deep sequencing datasets generated by St. Jude Children’s Research Hospital (St. Jude), HudsonAlpha Institute of Biotechnology (HAIB), and WuXiNextCode and whole-exome sequencing datasets generated by Broad Institute (BI) and Baylor College of Medicine (BCM) on five different Illumina sequencing platforms (Additional file 1: Table S1). To determine the lowest frequency at which a true somatic mutation can be distinguished from a sequencing error and to determine site-specific sequencing error rates, we performed a dilution experiment using a matched cancer/normal cell line COLO829/COLO829BL (ATCC CRL-1974 and ATCC CRL-1980), both of which were established from the same patient: COLO829 was from malignant melanoma and COLO829BL was from the matching normal lymphoblastoid. We targeted known somatic substitution mutations [20, 21] by amplicon sequencing (size of 130 ~ 170 bp) on an Illumina HiSeq 2500 sequencer (abbreviated as HiSeq).

We next compared the effect of polymerases by using Q5 and Kapa polymerases to generate amplicon libraries (“Methods”), which were sequenced on the latest Illumina sequencing platform NovaSeq 6000 (abbreviated as NovaSeq) at both St. Jude Children’s Research Hospital and HudsonAlpha Institute of Biotechnology sequencing centers (see Additional file 1: Table S1). To study the effect of sample-level damages, a high-depth sequencing (~ 50,000X coverage) dataset generated by hybridization-capture of 47 leukemia samples (*manuscript in preparation*) was used. To ascertain enrichment PCR errors, this hybridization-capture dataset was also compared with an aggregated whole-genome sequencing dataset. To evaluate the broad applicability of our observed error profiles in additional sequencing centers, the whole-exome sequencing (WES) data generated by Broad Institute and Baylor College of Medicine (the two TCGA sequencing centers) were used.

### Substitution error measurement

To measure substitution error, we took advantage of the high-depth sequencing data generated from the flanking sequences in the amplicons known to be devoid of genetic variations. Specifically, the substitution error rate for a given genomic site *i* was measured as follows:

$$ {\mathrm{error}\ \mathrm{rate}}_i\left(g>m\right)=\frac{\#\mathrm{reads}\ \mathrm{with}\ \mathrm{nucleotide}\ m\ \mathrm{at}\ \mathrm{position}\ i}{\mathrm{Total}\#\mathrm{reads}\ \mathrm{at}\ \mathrm{position}\ i} $$

where *g* indicates the reference allele at genomic locus *i* and *m* represents each of the three possible substitutions caused by sequencing error. For example, at a given site with reference allele A, we can calculate error rates for the three possible mismatches A>C, A>G, and A>T, respectively. Please note that although the nomenclature “error rate” implies that the measured subject is caused by noise and the nomenclature “mutant allele fraction” (MAF) implies that the measured subject is a true somatic mutation, we use both nomenclatures interchangeably in this paper because they have the same formula.

### Establishing the benchmark dataset

To investigate error profiles and the limits of variant detection, we established a truth dataset composed of 19 somatic single-nucleotide variants (SNVs) from the matched cancer/normal cell lines COLO829 and COLO829BL, which were derived from the same patient [21]. To benchmark the variant detection limit, we spiked-in 0.1% and 0.02% of COLO829 (cancer) genomic DNA into COLO829BL (normal) genomic DNA, resulting in two specimens diluted at 1:1000 and 1:5000, respectively, each with two replicates. The cancer and normal cell lines were also sequenced at 30,000X and 50,000X (Additional file 1: Table S2a–c), respectively, to validate the wildtype status of sequences flanking the target SNVs in the cell lines. More importantly, the undiluted cancer cell line data allowed us to characterize false-positive detections from 1:1000 and 1:5000 dilution datasets because the mutant allele fraction of a false-positive call would not exhibit 1000- to 5000-fold increase in the undiluted cancer cell line. By plotting MAF in diluted versus undiluted samples of every position on the 18 amplicons (Additional file 2: Figure S1), we found that the only sites exhibiting this pattern of MAF increase were the 18 targeted variants. Therefore, we conclude that no additional somatic variants exist in the 18 amplicons that we analyzed. The target SNVs were selected by accounting for the genomic aneuploidy at chromosome 1q, which exhibits loss-of-heterozygosity (LOH) and has four copies [20, 21] in the cancer cell line (Additional file 2: Figure S2a). Our selected somatic SNVs included those with mutant alleles on 4 of 4, 2 of 4, or 1 of 4 copies of 1q (Additional file 2: Figure S2b; Additional file 1: Table S2a-c), resulting in six distinct MAF levels (i.e., 0.01%, 0.02%, 0.04%, 0.05%, 0.1%, and 0.2%) over the two dilutions. HiSeq amplicon sequencing was carried out at respective depths of 300,000X and 1000,000X for the 1:1000 and 1:5000 dilution samples. We note that the allele fractions of the germline variants remain ~ 0.5 in our dilution experiment because we used matched tumor/normal cell lines from the same individual.

### Identification of low-quality reads

In the HiSeq data, 92% of sequenced bases had a base quality score ≥ 30 (Additional file 2: Figure S3a,e,i)—that is, the estimated error rate was less than 0.1%. Reads were preprocessed (“Methods”) by trimming 5 bp at both ends of each read (Fig. 1b; Additional file 2: Figure S3b, f, j; (“Methods”) to remove potentially low-quality bases and possible adapter contamination. Reads with low-mapping quality were also removed from further analysis [22] (Additional file 2: Figure S3c, g, k). We evaluated the association between the overall read quality and error rates of the remaining reads. The overall read quality was measured as the total number of low-quality bases (defined as having a quality score ≤ 20, corresponding to an error rate of ≥ 1%) per read, and the error rate was measured by using the flanking bases in the amplicons as described above. Interestingly, approximately 50% of reads contained no low-quality bases and had an overall error rate of 0.02%, and approximately 1% of reads contained ≥ 16 low-quality bases and had an error rate of 0.08% (Fig. 1c; Additional file 2: Figure S3d, h, l). Therefore, we defined low-quality reads (LQReads) as those with poor mapping quality or ≥ 5 low-quality bases. LQReads constitute ~ 30% of all reads in our experiment (Fig. 1c; Additional file 2: Figure S3d,h,l), and the remaining reads were considered high quality.

We developed an in silico error suppression method, CleanDeepSeq, to identify and filter the LQReads prior to allele counting (“Methods”). CleanDeepSeq is functionally equivalent to standard pileup in terms of allele counting. Since the target fragment size could be short (such as the 130~170 bp in our amplicon dataset), the forward and reverse reads in a paired-end sequencing setting may have significant overlaps. CleanDeepSeq was also designed to account for the concordance between forward and reverse readouts so that discordant readouts were not counted and concordant readouts were counted only once (“Methods”).

### Comparison with standard pileup

**Fig. 2:**
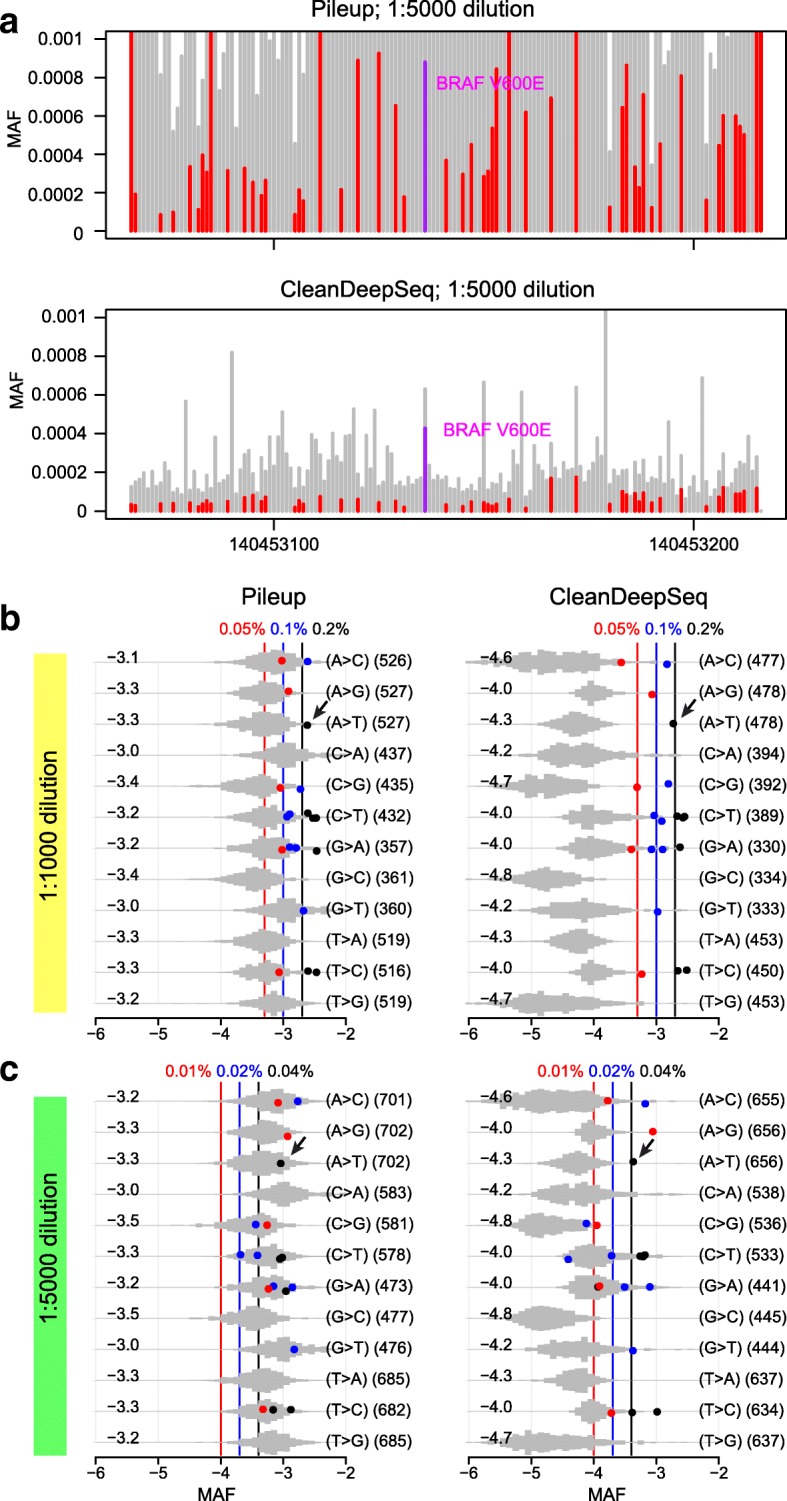
Comparison of sequencing errors with known somatic mutations in deep sequencing data generated from diluted COLO829 cancer cell line. **a** Error rate (*y*-axis) in *BRAF* V600 amplicon (*x*-axis: chr7 positions) under standard pileup (top) and CleanDeepSeq (bottom). A>T errors are shown in red and other errors shown in gray. Known somatic mutation *BRAF* V600E is shown in purple. Also shown are error rates summarized at sample level by pileup (left panels, “Methods”) or CleanDeepSeq (right panels) for 1:1000 dilution (**b**) and 1:5000 dilution (**c**). The 12 possible substitution patterns (first parenthesis) are depicted in rows. Median error rates (log10 scale) are indicated on the left, and sample sizes (number of genomic sites) for the histogram are indicated on the right in the second parenthesis. The *x*-axis displays the error rate in log10 scale. The designed MAF ladders for the known somatic mutations were depicted using red, blue, and black lines labeled on top, and the known somatic mutations were colored according to their expected MAF. Black arrow: *BRAF* V600E, which has 4 mutant alleles and 2 wildtype alleles in COLO829, so that at 1:1000 dilution and 1:5000 dilution the expected MAF are 0.002 and 0.0004, respectively (“Methods”)

We first compared our CleanDeepSeq method with the standard pileup method because both algorithms are designed to perform allele counting from aligned reads (such as from bam files), which serves as the starting point for most current mutation callers. As shown in Fig. 2a, the A>T error rate is dramatically suppressed from ~ 10^−3^ (by standard pileup method; top panel) to 0.5 × 10^−4^ (by CleanDeepSeq; bottom panel), rendering the *BRAF* V600E variant (with nucleotide change A>T) easily separated from background sequencing errors. At the sample level, we observed a median error rate of 0.4 × 10^−3^ to 1.0 × 10^−3^ for the 12 substitution patterns by using standard pileup on data (“Methods”) generated from two dilution samples (Fig. 2b, c, left panels), which is consistent with previous reports [1]. Consequently, somatic mutations (solid-color dots in Fig. 2b, c) with MAF < 0.002 cannot be distinguished from sequencing errors (gray-color histograms in Fig. 2b, c). By contrast, application of CleanDeepSeq resulted in a > 10-fold reduction in error rates (median error rate 0.2 × 10^−4^ to 1.0 × 10^−4^ in both dilutions), which clearly discriminates the MAF of true somatic variants from sequencing errors for most somatic mutations, including *BRAF* V600E (Fig. 2b, right panel). The same results were observed in the replicate experiments (Additional file 2: Figure S4–S5). Therefore, in the following experiments, we present only data filtered by CleanDeepSeq unless otherwise noted.

**Fig. 3:**
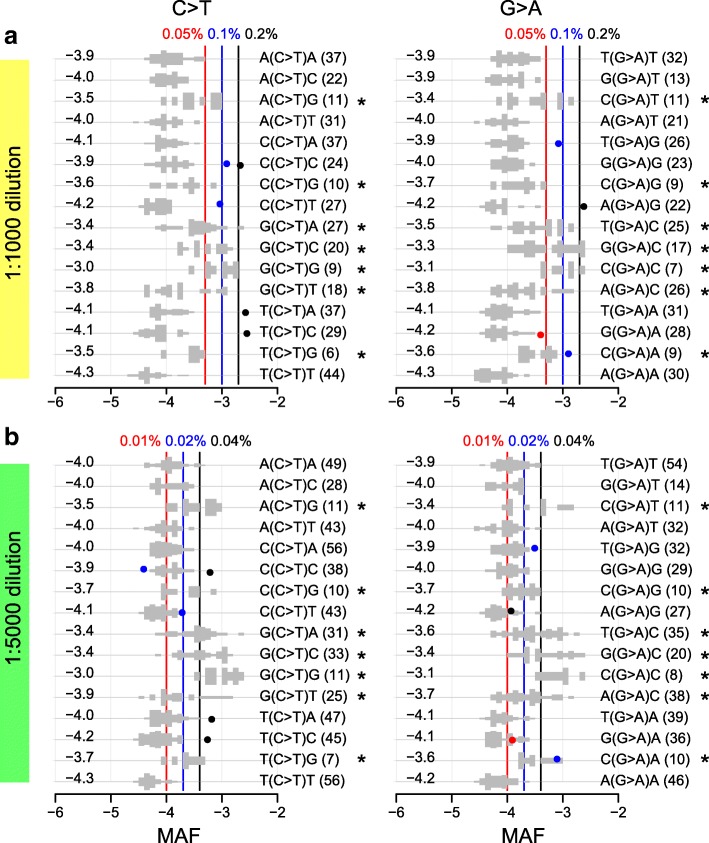
Context dependency of C>T/G>A errors in deep sequencing data generated from diluted COLO829 cancer cell line. C>T (left panels) and G>A (right panels) errors are decomposed into 16 contexts by including one 5′ base and one 3′ base for 1:1000 dilution (**a**) and 1:5000 dilution (**b**), respectively. Contexts showing elevated error rate are marked with an asterisk “*”. See Fig. 2 for legends

Different substitution patterns had different error rates. Specifically, C>T/G>A change had the highest error rate, with a median of ~ 10^−4^ in the CleanDeepSeq data—likely due to spontaneous deamination of methylated cytosine to uracil [1]. Consequently, C>T/G>A mutations remained indistinguishable from sequencing errors in the dilution series. To gain more insight into the error profile of C>T/G>A substitution (which is also the most common mutation type in cancers [23]), we performed signature analysis of C>T/G>A sequencing errors similar to mutational signature analysis in cancer [24]. The C>T errors exhibited a strong context dependency, with elevated error rates for G(C>T)N or N(C>T)G and the highest error rate in G(C>T)G (Fig. 3, left panels). As expected, this pattern was observed for G>A errors in a reverse complementary fashion (Fig. 3, right panels). Other substitution types did not exhibit sequence context dependency as strong as that of C>T/G>A (data not shown). Stratifying sequence mutations by their sequence context improved the precision for distinguishing somatic substitutions from sequencing errors in both 1:1000 and 1:5000 dilutions (Fig. 3; Additional file 2: Figure S4, S6).

### Comparison between sequencing centers, platforms, and DNA polymerases

Because NovaSeq offers much higher throughput for NGS data generation than HiSeq does, we performed amplicon sequencing (at both StJude and HAIB, see Additional file 1: Table S1) using the same COLO829 dilution samples and the same library preparation procedures. We found that NovaSeq has a comparable error profile to HiSeq across sequencing centers (Additional file 2: Figure S4, S7–S8). Interestingly, data from NovaSeq demonstrated a more homogeneous error profile than that of HiSeq, indicating an improvement at the sequencer level.

**Fig. 4:**
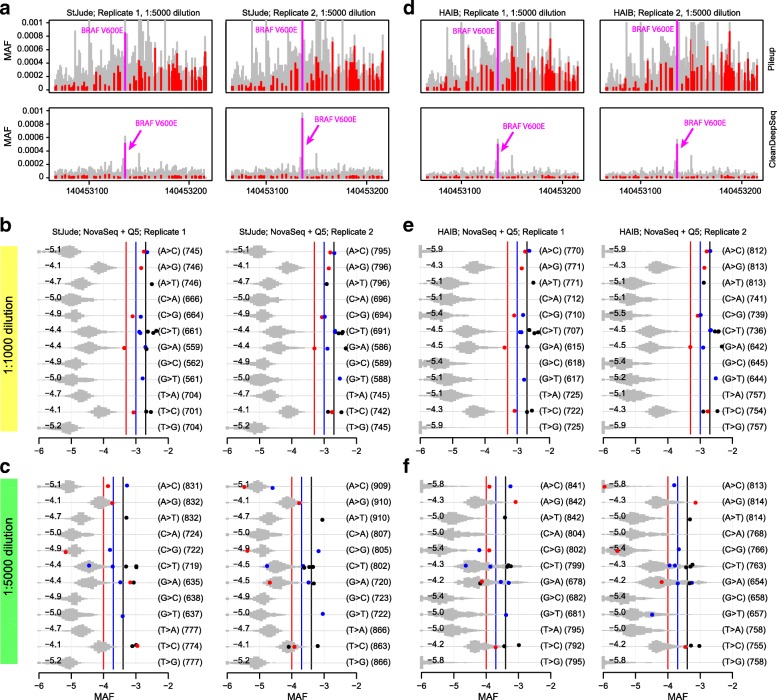
Error profile in NovaSeq + Q5 dataset generated by StJude (**a**, **b**, **c**) and HAIB (**d**, **e**, **f**). **a**, **d** Error rate (*y*-axis) in *BRAF* V600E amplicon (*x*-axis: chr7 positions) under direct pileup (top) and CleanDeepSeq (bottom). Also shown are error rates of the 12 change types across two dilutions: **b**, **e** 1:1000 dilution; **c**, **f** 1:5000 dilution, see Fig. 2 for legends

We next evaluated whether a different polymerase would affect the error profiles. Because libraries used for the HiSeq and NovaSeq datasets were prepared by using the Kapa DNA polymerase (“Methods”), we generated the NGS library with NEB Q5 polymerase, a high-fidelity enzyme, using the same COLO829 dilution samples, and sequenced the library on NovaSeq. As shown in Fig. 4 and Additional file 2: Figure S9, the C>T/G>A error rates were further suppressed by Q5, from 0.7 × 10^−4^ to 0.4 × 10^−4^, and changes including A>C/T>G, A>T/T>A, C>G/G>C, and C>A/G>T had error rates of ~ 10^−5^. As a result, Q5 enzyme library construction combined with NovaSeq would allow detection (assuming no sample-level DNA damage) of A>C/T>G, A>T/T>A, C>G/G>C mutations at levels < 0.01%; C>A/G>T mutations at a level of ~ 0.01%; C>T/G>A mutations in low error rate contexts at a level of ~ 0.01%; A>G/T>C mutations at a level of ~ 0.05%; and C>T/G>A mutations in high error rate contexts at a level of > 0.1% (Additional file 2: Figure S9). This observation was consistent between StJude and HAIB datasets (Fig. 4 and Additional file 2: Figure S9), demonstrating the reproducibility of this error profile.

Our data also indicated that “forced calling” of hotspot mutations without considering error rate may result in false-positives. For example, hotspot mutation *BRAF* K601E is a T>C change at chr7:140453134 (hg19; Additional file 2: Figure S10), which was detected in > 100 tumors in COSMIC database [25]. This site has an allele fraction of ~ 0.0003 in two replicates of both 1:1000 and 1:5000 dilutions across StJude and HAIB datasets, making it tempting to call as a true mutation. However, because the undiluted cancer sample does not show a corresponding elevation of allele fraction (~ 0.0002; Additional file 2: Figure S10), it is apparently a false-positive call. In fact, it is clear from Fig. 4b, c, e, f that T>C changes had a much-elevated median error rate of ~ 10^−4^ even after error suppression by CleanDeepSeq. Therefore, our error-profile analysis may help reduce the incidence of such false-positive calls.

To determine the accuracy of our error model at lower sequencing depths, we downsampled our NovaSeq + Q5 dataset (generated by StJude) to an actual depth of 40,000X–50,000X (Additional file 2: Figure S11). Even at these lower sequencing depths, the known mutations are still clearly separated from background sequencing errors.

### Error rate distribution of cancer-related substitutions and hotspot substitutions

We asked how the above results may influence the sensitive detection of cancer mutations. We found that 28.2% (Additional file 1: Table S3a) of the somatic SNVs listed in COSMIC [25] (v82; mostly adult cancers) are C>T/G>A mutations in high error rate contexts. To account for potential germline variants present in the COSMIC database, variants with a population allele fraction > 0.1% (defined by the ExAC database [26]) were removed. We found that 28.3% of COSMIC variants are in high error rate contexts. Interestingly, if a requirement of recurrence in ≥ 10 patients is added, only 16.5% of COSMIC variants are in high error rate contexts. For pediatric cancers [15], 20.8% of somatic mutations (8% for neuroblastoma; Additional file 1: Table S3a) are C>T/G>A mutations in high error rate contexts. These results collectively indicated that > 70% of the somatic substitutions are in low error rate contexts and that high-depth sequencing analysis can detect them at low (0.01 ~ 0.1%) frequency. Similarly, by using the list of hotspot substitutions defined by Taylor and colleagues [27], we found that 73% (Additional file 1: Table S3b) of hotspot substitutions are in low error rate contexts and high-depth sequencing analysis can detect them at low (0.01 ~ 0.1%) frequency.

### Errors introduced by specimen handling and/or storage

**Fig. 5:**
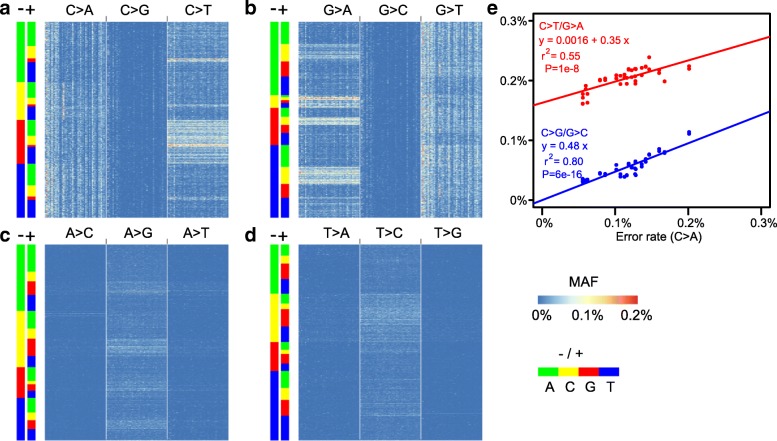
Sample-specific errors in high-depth capture sequencing data. Each column represents a leukemia sample (in total 47 samples) while each row represents a genomic position that was sequenced in all samples. The genomic positions were assigned to panels **a**–**d** by the nucleotide at corresponding positions, i.e., C at (**a**), G at (**b**), A at (**c**), and T at (**d**) as heatmaps. In each panel, MAF for all three possible substitution types were shown in three groups indicated at the top of each panel, sorted by their neighboring DNA context (i.e., 3′ (−) or 5′ (+) flanking bases). Vertical patterns show the sample-level DNA damage which is apparent in C>A and G>T mutation. **e** Significant correlation of sample-specific error (surrogated by C>A error rate) with error types C>T/G>A and C>G/G>C but not for other type (data not shown). The linear regression and r-squared values are indicated

To investigate sample-level errors, which may indicate specimen handling/storage issues, we analyzed a hybridization-capture dataset of 47 samples (“Methods”). By using CleanDeepSeq, we generated a heatmap to show the sequencing error rate in each sample (columns) stratified by sequence context associated with each substitution pattern (rows). As shown in Fig. 5a, b, C>T/G>A errors exhibited a horizontal pattern across all samples, replicating the context dependency observed in the COLO829 dataset (Fig. 3; Additional file 2: Figure S4). By contrast, C>A/G>T errors exhibited a vertical (i.e., sample-specific) pattern regardless of sequence context, which may be attributable to sample-specific 8-oxoG stress [1, 17, 18].

We then investigated whether sample-specific DNA damage (imputed by using C>A/G>T substitution as a surrogate) could account for other types of sequencing errors. Indeed, we found that the C>A error rate was significantly correlated with that of C>G/G>C (linear regression *P* value = 6 × 10^−16^) and C>T/G>A (linear regression *P* value = 10^−8^) (Fig. 5e), indicating that sample-specific DNA damage also contributes to an elevated error rate of C>G/G>C and C>T/G>A changes.

### Broad applicability of CleanDeepSeq

We analyzed whole-exome sequencing data generated by the Broad Institute and Baylor College of Medicine Human Genome Sequencing Center, the two sequencing centers for the Cancer Genome Atlas (TCGA) project and the NCI’s Therapeutically Applicable Research To Generate Effective Treatments (TARGET) project. Given the limited sequencing depth of whole-exome sequencing (100–200X), it is impossible to calculate site-specific error rates. Therefore, we focused our analysis on sample-level error rates (“Methods”). We analyzed the neuroblastoma whole-exome sequencing dataset [28] generated by the Broad Institute (“Methods”) because it is known that library preparation artifacts were introduced during DNA-shearing of the library construction process by high-energy sonication, resulting in the oxidation of guanine bases (8-oxoG). It was reported previously that 8-oxoG artifacts causing elevated C>A changes were present in the Exome_Native dataset (i.e., no whole-genome amplification) but not in the Exome_WGA (i.e., library prepared using whole-genome amplified DNA) dataset [28].

**Fig. 6:**
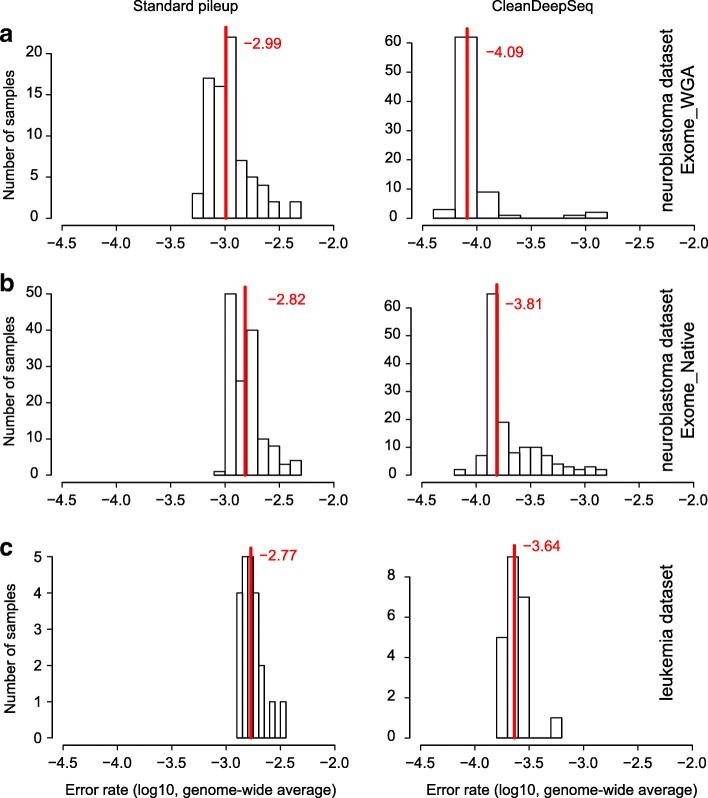
Genome-wide average error rate in neuroblastoma datasets (panels **a**, **b**) and an AML dataset (panel **c**). Shown are histogram of genome-wide average error rate (“Methods”) by standard pileup (left panels) and CleanDeepSeq (right panels). In the neuroblastoma dataset (generated by Broad Institute, “Methods”), the Exome_Native subset (**b**) is known to have sample-level damages while the Exome_WGA subset (**a**) does not have sample-level damages. Also included are an AML dataset (generated by Baylor College of Medicine) (**c**). Red vertical lines and numbers indicate median

Using standard pileup, the error rate of both Exome_WGA and Exome_Native is ~ 0.1% (log10 scale of − 3 in the left panel of Fig. 6a, b), consistent with previous reports [1, 10, 11]. Applying CleanDeepSeq resulted in a 10-fold reduction of error rate (~ 0.01%, log10 scale of − 4 in the right panels of Fig. 6a, b) in both datasets. Interestingly, the Exome_Native dataset had a slightly higher error rate than the Exome_WGA dataset did by both standard pileup and CleanDeepSeq (Fig. 6a, b), which is consistent with the known sample-level damage in the Exome_Native dataset. The WES data generated by Baylor College of Medicine Human Genome Sequencing Center [15] were from leukemia samples (“Methods”), and we also found a 10-fold reduction in error rate of CleanDeepSeq compared to that of standard pileup here (Fig. 6c). Together, these results provided corroborating evidence of the power of error suppression by CleanDeepSeq.

### Second-enrichment PCR errors

**Fig. 7:**
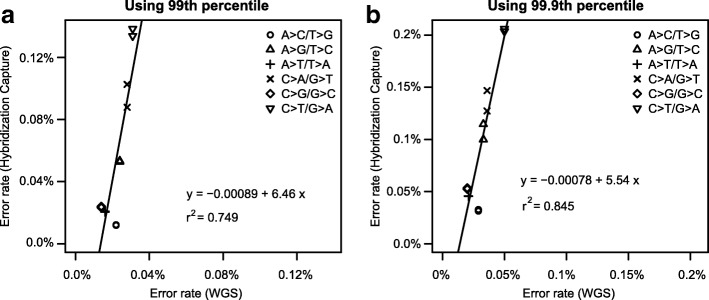
Error rate comparison between hybridization-capture and aggregated WGS datasets. Summary statistics (“Methods”) are calculated with 99th percentile (*P* = 3 × 10^−4^; **a**) and 99.9th percentile (*P* = 2 × 10^−5^; **b**). We also tried 90th percentile but the linear fitting is poor (*r*^2^ = 0.47; slope = 4.4; data not shown) due to the fact that many loci have MAF of 0 as described in the “Methods” section

We next studied the errors introduced by enrichment PCR (6–18 cycles). For this purpose, we aggregated the sequencing data of 1663 whole genomes [29] (“Methods”) that had undergone first-enrichment PCR. The hybridization-capture sequencing dataset, which underwent two enrichment PCR rounds, was compared to WGS dataset with CleanDeepSeq. Both datasets were sequenced by using Illumina X Ten. We found a statistically significant linear relationship between hybridization-capture targeted sequencing data and WGS data among the 12 error types, and a ~ 5.5- to 6.5-fold increase in errors was observed in capture sequencing data (Fig. 7).

## Discussion

In the past decade, rapid progress in NGS has dramatically shifted the paradigm of biomedical research and NGS is now quickly moving into clinical practices. However, the general perception of high error rate (> 0.1%) in conventional NGS data has hindered its application in detecting low-frequency variants. A comprehensive understanding of the sources of errors introduced in the NGS workflow is, therefore, key to further improving sequencing accuracy.

Our analyses uncovered several sources of errors. We found that error rates have substitution-type and sequence context dependencies, which reflect fidelity of DNA polymerases. We also found that C>A/G>T errors are enriched in a subset of samples, which indicates sub-optimal handling/storage conditions. Because sample handling was not documented in the present study, future studies designed with controlled experiments are warranted to study the optimal handling/storage conditions required to minimize errors. However, despite the significant improvements to overall error rate introduced by removal of LQReads, the A>G/T>C errors remain high. Further enzymatic optimization or DNA repair treatments during library construction might resolve these issues but are out of the scope of this work.

This study was focused on error profiling but not variant detection, although error suppression will ultimately improve variant detection. Variant detection can be formulated into three related but distinct study designs. First, one may have a case-control design, where the sample of interest is compared against a control sample. Indeed, a simple combination with the existing deepSNV algorithm (which assumes case-control design) [30] resulted in significant reduction (3- to 6-fold) of false positives by CleanDeepSeq as compared to the standard pileup algorithm, without compromising sensitivity (Additional file 2: Figure S12). Using the mutation caller MuTect [31], we also found that use of CleanDeepSeq resulted in 3- to 30-fold reduction of false positives compared with standard pileup without compromising sensitivity (Additional file 2: Figure S13). Second, one may have a cohort design where multiple samples are sequenced simultaneously and each sample is compared individually against the remaining samples (“aggregate control”, see [32]). However, the recent discovery of ubiquitous AML-associated mutations [33] in peripheral blood from healthy adults pose significant challenges in selecting control samples that are truly absent of low-frequency mutations. Such unrecognized low-frequency variation may lead to false-negative results in a design with controls. Third, it is therefore desirable to perform variant detection in the sample of interest without a control. In fact, the data presented in this work indicate the feasibility of performing single-sample variant detection, which we are currently researching.

## Conclusions

Our results provided important insights on further improving sequencing error rates in future.

## Methods

### Amplicon sequencing of diluted COLO829 cell line

COLO829BL and COLO829 DNA was extracted by using the DNeasy Blood & Tissue Kit (Qiagen), and the mixture DNA samples were generated by spiking-in 0.1% and 0.02% of COLO829 into COLO829BL. Primers for SNV targets (Additional file 1: Table S2d) sized 130 bp to 170 bp were designed by using Primer3. PCR was performed with the KAPA HiFi HotStart ReadyMix PCR Kit and NEBNext Q5 Hot Start HiFi PCR Master Mix, 10 μM of each primer, 50 ng of COLO829BL, COLO829, two replicates of 0.1% mixture, and two replicates of 0.02% mixture DNA for each target by using the following PCR conditions: 95 °C for 5 min, 26 cycles of 98 °C for 20 s, 63 °C for 15 s, 72 °C for 15 s, and 72 °C for 1 min before storage at 4 °C (Kapa HiFi HotStart); 98 °C for 30 s, 26 cycles of 98 °C for 10 s, 65 °C for 15 s, 72 °C for 20 s, and 72 °C for 2 min before storage at 4 °C (NEBNext Q5). All amplicons were quality-checked on a 2% agarose E-gel (Invitrogen), then pooled in bins and purified by Agencourt Ampure XP Beads. A total of 100 ng of each pooled amplicon was end-repaired, adapter-ligated, and enriched via 8 cycles of PCR by using either KAPA HiFi HotStart ReadyMix PCR Kit or NEBNext Q5 Hot Start HiFi PCR Master Mix. Finally, amplicon libraries were pooled by specific ratios to enable generation of 300,000X coverage for 0.1% spike, 1000,000X coverage for 0.02% spike, 50,000X for COLO829BL, and 30,000X coverage for COLO829 on the Illumina HiSeq 2500 Rapid mode and NovaSeq 6000 S1 flow cell paired-end 2 × 101 cycles sequencing.

### COLO829 dataset

Melanoma cell line COLO829 lost heterozygosity in 1q with 4 copies [21] (Additional file 2: Figure S2a), and its matching normal cell line COLO829BL had a diploid genome. In 1q, there are 3 groups of SNVs with different numbers of MAFs (Additional file 2: Figure S2b): 100% (4 of 4 total alleles); 50% (2 of 4 total alleles); and 25% (1 of 4 total alleles). We took advantage of this fact and selected 16 SNV markers from the 1q region: 6 SNVs with MAF of 1.0, 7 SNVs with MAF of 0.5, and 3 SNVs with MAF of 0.25. We also selected 2 SNVs from a diploid region in chr4 (so we would have 5 SNVs with 1 mutant allele in each cancer cell). We also selected *BRAF* V600E, an oncogenic hotspot mutation detected in this sample that has a MAF of 0.67 (4 of 6 copies are mutated), totaling 19 SNVs (Additional file 1: Table S2d). Marker chr1.203055000. G>A failed the Q5 amplicon, so there are 18 SNVs in the NovaSeq + Q5 dataset.

The expected number of MAFs is calculated as *MAF* = *a*/(1 × 4 + 1000 × 2) ≈ *a*/2000 for the 1q markers with 1:1000 dilution, *MAF* = 1 × *a*/(1 × 4 + 5000 × 2) ≈ *a*/10000 for the 1q markers with 1:5000 dilution, where *a* (= 1, 2, 4) represents the total number of mutant alleles in 1 cancer cell for a given SNV. A similar approximation was used for *BRAF* V600E (*a* = 4) and the 2 SNVs (*a* = 1) in chr4. The red, blue, and black vertical lines in Figs. 2, 3, and 4 (and Additional file 2: Figure S5–9, 11) correspond to *a* (=1, 2, 4) for corresponding dilution concentrations, respectively.

### Hybridization-capture dataset

Genomic DNA was sheared to ~ 150- to 200-bp average size by using a Covaris LE220 focused ultrasonicator. The fragmented DNA was then end-repaired, dA-tailed, adapter-ligated, and enriched by PCR amplification using Kapa HTP library preparation kit Illumina 96rxn. Designed baits were hybridized with adapter-ligated DNA libraries for 64 to 72 h. Then, the bait-target hybrids were captured by streptavidin beads and enriched via secondary PCR enrichment. The capture libraries were sequenced by performing paired-end 150 cycles on the Illumina HiSeq X Ten system at 50,000X. This dataset has a median of 87,094 (range 31,437–129,934) base pairs covered at ≥ 15,000X across 47 samples (see detailed sample list in Additional file 1: Table S4).

### WGS sequencing

DNA was extracted from stored samples by using either the QIAamp DNA Blood Mini Kit (QIAGEN cat#51106) or the DNeasy Blood & Tissue Kit (cat# 69506). After extraction, the DNA concentration was fluorometrically measured by using the Quant-iT dsDNA Assay Kit (Life Technologies cat#Q33130), and DNA integrity was verified visually by agarose gel electrophoresis (E-Gel, Life Technologies, cat#G8008-01). Whole-genome sequencing (WGS) was performed at the HudsonAlpha Institute for Biotechnology Genomic Services Laboratory (Huntsville, AL, USA) by using Illumina HiSeq X Ten sequencers. A total of 1663 whole-genome samples from a previous St. Jude LIFE (SJLIFE) study [29] (see detailed sample list in Additional file 1: Table S5) were included in this work.

Whole-genome sequencing data were also analyzed by using CleanDeepSeq for each sample. To account for polymorphisms, within each sample, only loci with ≥ 20X coverage and > 95% (so that binomial *P* value of observing 1 non-reference alleles from 20 reads is 4 × 10^−5^ and binomial *P* value of observing 2 non-reference alleles from 40 reads is 1.5 × 10^−9^ given the locus is heterozygous) reads being reference allele were merged into a single-count file. Loci with heterozygous calls (i.e., no alleles with fraction > 95%) in any subject were excluded from analysis. We used only loci with ≥ 20,000X collapsed coverage in our error analysis for this dataset.

### Direct pileup

To compare CleanDeepSeq with direct pileup (Fig. 2b), we implemented the command “lofreq plpsummary -Q 30 -q 30 -m 55 -d100000000” from LoFreq [34], which means to count bases (both reference and non-reference alleles) by using a quality cutoff of 30 and include only reads with mapping quality (MAPQ) > 55 (value 255 also discarded because it indicates that the mapping quality is not available (https://samtools.github.io/hts-specs/SAMv1.pdf)). Consistent with a previous report [35], recalibration [36] did not significantly change the result of pileup (data not shown). Direct pileup on NovaSeq resulted in an error rate ~ 10^−4^, indicating a significant improvement of the sequencer. However, CleanDeepSeq improved (10-fold fewer errors) error suppression beyond pileup for changes including A>C/T>G, A>T/T>A, C>A/G>T. However, the direct pileup by LoFreq generated irregular counts when the depth exceeded 10 million; therefore, we downsampled the raw data to 20% for NovaSeq experiments.

### Neuroblastoma whole-exome sequencing dataset with known sample damage and AML whole-exome sequencing dataset

To study samples with known DNA damage, we downloaded a neuroblastoma whole-exome sequencing dataset [28] generated by Broad Institute in 2010 and 2012 (using Illumina sequencer GAII or HiSeq 2000; see “Methods”). This whole-exome sequencing dataset (76-bp paired-end) included native (Exome_Native) and whole-genome–amplified DNA (Exome_WGA) samples, of which the former were known to harbor elevated levels of C>A/G>T errors (Additional file 2: Figure S2 of Pugh et al. [28]) due to high-energy sonication at the DNA-shearing step during library construction. Only germline samples were analyzed. We counted alleles at every genomic site with CleanDeepSeq or lofreq as described above and utilized the 75-mer mappability track from UCSC genome browser (see below).

Because we are interested in sample-level DNA damage, we wanted to obtain sample-specific and site-specific error rates. However, because the sequencing depth is only 100X to 200X, we could not properly calculate the site-specific error rate for this dataset. We, therefore, focused on all well-covered sites (≥ 50X and with a dominant reference allele with fraction > 95%, so that the binomial *P* value of observing 1 non-reference allele from 50 reads is 4 × 10^−14^ and the binomial *P* value of observing two non-reference alleles from 50 reads is 1 × 10^−12^ given the locus is heterozygous) to calculate sample-level error rate (defined as total mismatch bases divided by total mapped bases, see [37]). An AML whole-exome sequencing dataset [15] (22 germline samples) generated by Baylor College of Medicine Human Genome Sequencing Center in 2012 (using Illumina sequencer HiSeq 2000; see “Methods”) was similarly anlayzed to further strengthen our conclusions.

### Application of deepSNV and MuTect to low-level substitution detection

To apply deepSNV (version 1.26.0) algorithm [30], we split our CleanDeepSeq counts of A, C, G, T at each site into halves—one for the reference strand and the other for the reverse strand—because deepSNV requires strand-specific counting. We then supplied the count data (dilution data as “case” and normal data as “control”) to the function “deepSNV” in R (version 3.4.4). Mutations were called with a *P* value cutoff of 0.05 after Bonferroni correction.

To apply the MuTect algorithm (version 1.1.4) [31], we implemented the following command: java -Xmx2g -jar muTect-1.1.4.jar --analysis_type MuTect --reference_sequence REFERENCE.fasta --input_file:normal GERMLINE.bam --input_file:tumor TUMOR.bam --out CALL_STATS.txt --coverage_file COVERAGE.wig --cosmic COSMIC.vcf --dbsnp DBSNP.vcf --downsampling_type NONE --force_alleles --tumor_f_pretest 0.000001 --gap_events_threshold 1000 --fraction_contamination 0.00, where GERMLINE.bam is our undiluted normal cell line and TUMOR.bam is (1) 1:1000, (2) 1:5000 diluted cell line, or (3) the undiluted cancer cell line. We applied MuTect to the NovaSeq + Q5 dataset generated by StJude. The initial run of MuTect generated irregular allele counts for the candidate markers that turned out to be due to the default behavior of MuTect to downsample the reads (stated as “The principle of this downsampling type is to downsample reads to a given capping threshold coverage. Its purpose is to get rid of excessive coverage, because above a certain depth, having additional data is not informative and imposes unreasonable computational costs.” in MuTect documentation from https://software.broadinstitute.org/gatk/documentation/tooldocs/3.8-0/org_broadinstitute_gatk_engine_CommandLineGATK.php). When this behavior is turned off (by adding the parameter “--downsampling_type NONE”), we cannot run MuTect—even at 20-Gb memory request—because of our data’s high depth. Therefore, we downsampled our bam file to 50,000X depth for each of the 18 amplicon regions so that we could run the MuTect algorithm. To test the improvement of MuTect variant detection accuracy by error suppression using CleanDeepSeq, we filtered the low-quality reads (as described in next section “Error suppression by CleanDeepSeq”) and created new bam files (both dilution dataset and normal dataset) as input for MuTect.

### Error suppression by CleanDeepSeq

Because the base quality dropped at read ends for HiSeq data (Fig. 1b; Additional file 2: Figure S3b, f, j), we trimmed the first and last five base pairs of the reads. This trimming would also clean up potential residual adapter/primer sequences. The same parameter is used for other datasets as well. To avoid artifacts attributable to mapping ambiguity, we used a stringent mapping quality (MAPQ) cutoff of 55 (value 255 also discarded because it indicates that the mapping quality is not available (https://samtools.github.io/hts-specs/SAMv1.pdf)), which affected 18.2% of reads (16.2% if using a MAPQ cutoff of 30; Additional file 2: Figure S3c, g, k) in the HiSeq dataset. Furthermore, because reads with insertion/deletions and/or structural rearrangements may introduce alignment ambiguity, we only included reads with substitution mismatches (i.e., the CIGAR string matches the regular expression /^\d + M$/; affecting ~ 1% reads; Additional file 2: Figure S3d, h, l). Reads with ≥ 5% bases of Phred quality score < 20 were also suppressed because they have elevated error rates (Fig. 1c; Additional file 2: Figure S3d, h, l). To avoid counting an allele from the same DNA fragment twice, we used the following procedure for fragments with overlapping read pairs: (i) if a base pair has only one readout in either forward or reverse read (non-overlapping part), it will only be counted as 1 if its Phred quality score is ≥ 30; (ii) if a base pair has two readouts in both forward and reverse reads (overlapping part), it will be counted as 1 if forward and reverse readouts are concordant and both have Phred quality score ≥ 30 or if only one readout has Phred quality score ≥ 30.

### Deep sequencing data analysis

For high-depth data, sites that were sufficiently covered (> 500X) and had a dominant allele (frequency > 95%) were counted. For error rate analysis (such as in Fig. 2b), we used 500,000X as the depth cutoff for the COLO829 data; 15,000X for hybridization-capture data; and 20,000X for collapsed WGS data to account for sampling uncertainty and different designed depths. For context analysis, the flanking bases were also required to have a dominant allele with frequency > 95%. The implicit assumption of a 95% threshold is that the error rate rarely exceeds 5%. Due to the possible presence of true low-level SNVs (such as mosaic mutations) that are not recognized, this threshold might lead to slightly over-estimated background error rates. Therefore, we consider a 95% threshold to be conservative for our reported error rates (i.e., the true error rates could be even lower).

### Usage of summary statistics

Usually, summary statistics such as median/mean are used to represent population averages. With sufficiently high depth, such as in Fig. 2, median is a good summary statistic for our purpose. However, with reduced depth, such as in downsampling (Additional file 2: Figure S11), most genomic sites have MAF 0, rendering mean or median non-informative. As a result, we used higher percentiles, such as 99.9th percentile, to represent the population characteristics. Because such a statistic is much less robust (in terms of sampling uncertainty) than are mean or median, we required a sufficient number of sample points to use this statistic in this work. Specifically, for the hybridization-capture dataset (Fig. 5), we required that there be > 20,000 genomic sites for each of the 12 substitution types for a sample to be included in the analysis (21 of the 47 hybridization-capture samples passed this threshold and are included in Fig. 5). This requirement ensures that there are > 20 genomic sites with error rate above the 99.9th percentile for each of the 12 substitution types. One advantage of using 99.9th percentile is that it automatically implies a false-positive rate of 0.1% (i.e., 99.9% of genomic sites have lower allele fraction than this statistic). A similar reasoning was used for the comparison between hybridization-capture dataset and the whole-genome sequencing dataset (Fig. 7).

### Other analysis details

Reads were aligned by using bwa (0.7.12-r1039) with option “aln.” To avoid artifacts due to paralog mapping, we included only base pairs in uniquely mappable regions for 100-mers (http://hgdownload.soe.ucsc.edu/goldenPath/hg19/database/wgEncodeCrgMapabilityAlign100mer.txt.gz for hg19 and http://hgdownload.soe.ucsc.edu/gbdb/hg38/hoffmanMappability/k100.Umap.MultiTrackMappability.bw for hg38; downloaded March 2018) and for 75-mers (http://hgdownload.cse.ucsc.edu/goldenPath/hg19/encodeDCC/wgEncodeMapability/wgEncodeCrgMapabilityAlign75mer.bigWig). Only regions with a mappability score of 1 and length > 300 bp were considered. Furthermore, the first and last 50 bp of a region were excluded to account for potential edge effects.

## Additional files

Additional file 1: Supplementary Tables S1-S5. **Table S1**. Datasets used. Information provided includes data type, provider, analysis type, target region size, sequencing depth, dilution ratio, and sequencer. **Table S2a**. Designed 19 substitution markers in COLO829 experiment (HiSeq+Kapa Enzyme; StJude dataset). Listed are chromosome, position (hg19), mutation context, ploidy for each mutations. Also listed are the MAF, mutant allele counts (Mut), total coverage (Tot) for each replicate of each lane output for Normal, Tumor, and two dilutions (1:1000 and 1:5000). For ploidy, 4v4 means 4 out of 4 allels are mutated in cancer cells, 2v4 means 2 out of 4 allele are mutated; 1v4 means 1 out of 4 alleles are mutated; 4v6 means 4 out of 6 alleles are mutated; 1v2 means 1 out of 2 alleles are mutated. The allele counts by CleanLens are based on Phred score cutoff 38. *: BRAF V600E. **Table S2b**. Designed 19 substitution markers in COLO829 experiment (NovaSeq with Kapa enzyme; StJude dataset). Listed are chromosome, position (hg19), mutation context, ploidy for each mutations. Also listed are the MAF, mutant allele counts (Mut), total coverage (Tot) for each replicate of each lane output for Normal, Tumor, and two dilutions (1:1000 and 1:5000). For ploidy, 4v4 means 4 out of 4 allels are mutated in cancer cells, 2v4 means 2 out of 4 allele are mutated; 1v4 means 1 out of 4 alleles are mutated; 4v6 means 4 out of 6 alleles are mutated; 1v2 means 1 out of 2 alleles are mutated. The CleanLens allele counts based on Phred score cutoff 30. *: BRAF V600E. **Table S2c**. Designed 19 substitution markers in COLO829 experiment (NovaSeq with Q5 enzyme; StJude dataset). Listed are chromosome, position (hg19), mutation context, ploidy for each mutations. Also listed are the MAF, mutant allele counts (Mut), total coverage (Tot) for each replicate of each lane output for Normal, Tumor, and two dilutions (1:1000 and 1:5000). For ploidy, 4v4 means 4 out of 4 allels are mutated in cancer cells, 2v4 means 2 out of 4 allele are mutated; 1v4 means 1 out of 4 alleles are mutated; 4v6 means 4 out of 6 alleles are mutated; 1v2 means 1 out of 2 alleles are mutated. The CleanLens allele counts are based on Phred score cutoff 30. *: BRAF V600E. N.D: PCR failure. **Table S2d**, Primers for the 19 substitution markers for COLO829 experiment. **Table S3a**. Mutation counts in pediatric cancers (non-NBL and NBL) and adult cancers (COSMIC v82) are listed in columns C,D,E. For COSMIC data, we also excluded markers with population allele frequency (AF) >=0.1% (from ExAC database with TCGA samples subtracted), and required mutation recurrence (Rec) to be >=1 (columns F, M, S), >=5 (columns G, N, T), and >=10 (columns H, O, U). The number of C>T/G>A mutations in high error rate context for each group are listed in columns J-O, with percentages of high error rate contexts summarized in columns P-U. **Table S3b**. Analysis of sequence context of hotspot substitutions defined by Chang et al. (PMID: 29247016). In total 947 hotspot substitutions mutated in 5 or more samples (column C) are included. The gene name (column A), amino acid change (column B), genomic substitutions (column D) were extracted from the source paper. The mutational contexts were provided in columns E,F,G, in case multiple mutations can cause the same amino acid change. C>T/G>A mutations in high error rate contexts were indicated with orange color. **Table S4**. List of 47 hybridization capture samples. Related to Fig. 5 and Fig. 7. **Table S5**. List of 1663 whole genome samples. Related to Fig. 7. (XLSX 190 kb)

Additional file 2: Supplementary Figures S1-S13. **Figure S1**. Comparison of mutant allele fraction (MAF) in diluted samples (y-axis) and undiluted cancer cell line (x-axis). **Figure S2**. Copy-number status of cell line COLO829 and ploidy of the 19 selected substitutions in this work. **Figure S3**. Quality metrics of sequenced datasets. **Figure S4**. Heatmap of error profiles across sequencing providers, sequencers, PCR enzymes, replicates, and dilutions. **Figure S5**. HiSeq error profile under CleanDeepSeq. **Figure S6**. Context dependency of C>T/G>A errors in HiSeq data under CleanDeepSeq. **Figure S7**. NovaSeq+Kapa error profile under CleanDeepSeq. **Figure S8**. Context dependency of C>T/G>A errors in NovaSeq+Kapa dataset under CleanDeepSeq. **Figure S9**. Context dependency of C>T/G>A errors in NovaSeq+Q5 dataset under CleanDeepSeq. **Figure S10**. False-postive introduced by “forced calling”. **Figure S11**. Error profiles in downsampling of NovaSeq + Q5 dataset. **Figure S12**. Comparison of standard pileup and CleanDeepSeq by using deepSNV on dilution experiments. **Figure S13**. Comparison of standard pileup and CleanDeepSeq by using MuTect on dilution experiments. (DOCX 5524 kb)
