# Supplementary material for: Analysis of error profiles in deep next-generation sequencing data
Source: Genome Biol. 2019 Mar 14;20:50. doi: 10.1186/s13059-019-1659-6 (PMC6417284; doi:10.1186/s13059-019-1659-6)
Supplement: Supplementary file 2 — Supplementary Figures S1-S13. Figure S1. Comparison of mutant allele fraction (MAF) in diluted samples (y-axis) and undiluted cancer cell line (x-axis). Figure S2. Copy-number status of cell line COLO829 and ploidy of the 19 selected substitutions in this work. Figure S3. Quality metrics of sequenced datasets. Figure S4. Heatmap of error profiles across sequencing providers, sequencers, PCR enzymes, replicates, and dilutions. Figure S5. HiSeq error profile under CleanDeepSeq. Figure S6. Context dependency of C>T/G>A errors in HiSeq data under CleanDeepSeq. Figure S7. NovaSeq+Kapa error profile under CleanDeepSeq. Figure S8. Context dependency of C>T/G>A errors in NovaSeq+Kapa dataset under CleanDeepSeq. Figure S9. Context dependency of C>T/G>A errors in NovaSeq+Q5 dataset under CleanDeepSeq. Figure S10. False-postive introduced by “forced calling”. Figure S11. Error profiles in downsampling of NovaSeq + Q5 dataset. Figure S12. Comparison of standard pileup and CleanDeepSeq by using deepSNV on dilution experiments. Figure S13. Comparison of standard pileup and CleanDeepSeq by using MuTect on dilution experiments. (DOCX 5524 kb) [file 13059_2019_1659_MOESM2_ESM.docx]

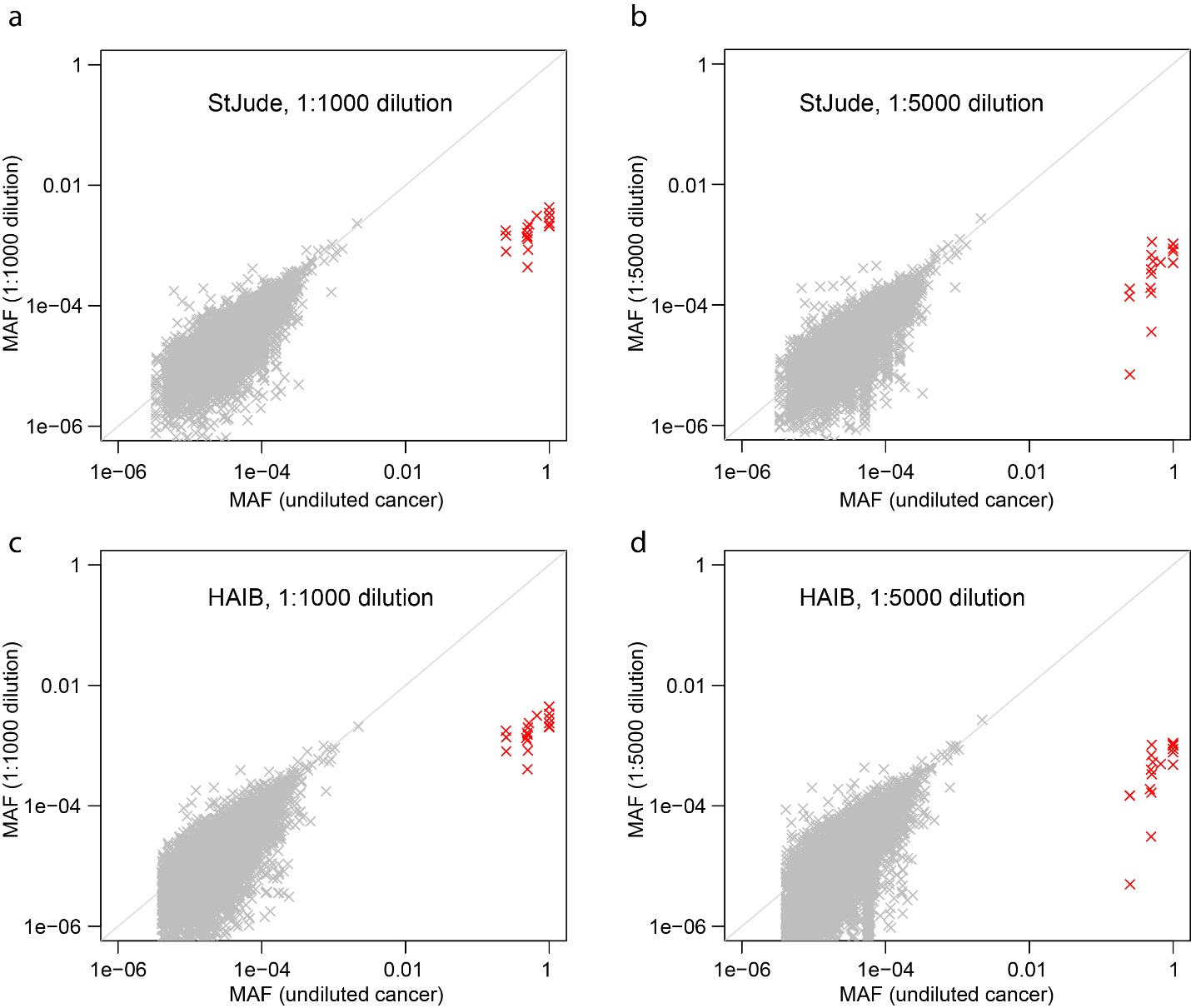


Figure S1. Comparison of mutant allele fraction (MAF) in diluted samples (y-axis) and undiluted cancer cell line (x-axis).

Data were generated by using NovaSeq+Q5 in StJude (panels **a**, **b**) or in HAIB (panels **c**, **d**). Allele counting was performed by using CleanDeepSeq. The 18 known somatic SNVs are highlighted in red.


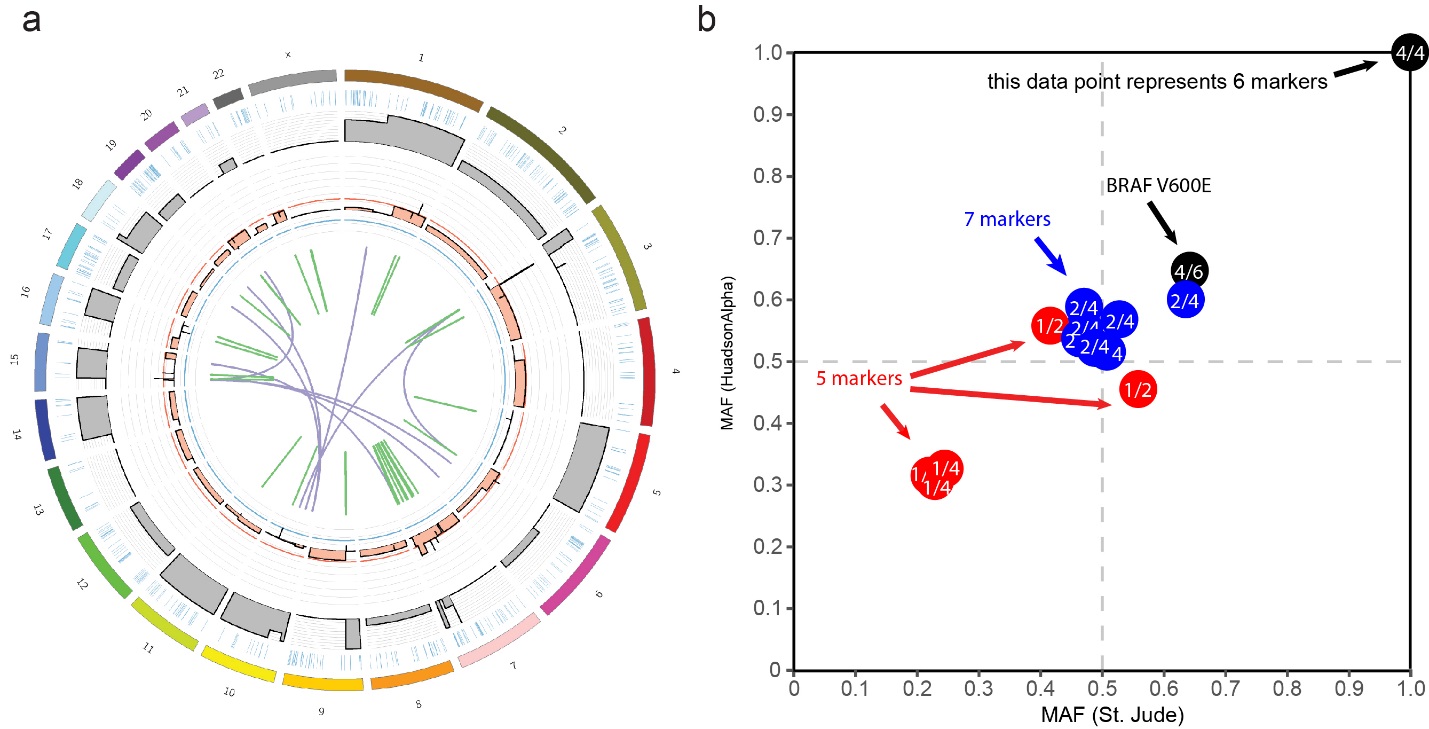


Figure S2. Copy-number status of cell line COLO829 and ploidy of the 19 selected substitutions in this work.

(a) The loss-of-heterozygosity (absolute B-allele fraction) difference between tumor and normal cells is shown as a gray track. Copy-number status is shown in the inner track, with gains shown with orange color toward the orange circle and losses shown with black color toward the blue circle. As supporting information, structural rearrangements are shown as lines and arches, with green indicating intra-chromosomal rearrangements and purple depicting inter-chromosomal rearrangements. Coding SNV/Indels are depicted in the first track inside the chromosomes. (b) MAF of the 19 selected markers from St. Jude whole-genome sequencing (x-axis) and HudsonAlpha whole-genome sequencing (y-axis). The markers are colored according to the total number of mutant alleles in each cancer cell (1: red; 2: blue; 4: black), which is indicated together with copy-number status in the corresponding locus. For example, “2/4” indicates that there are 4 copies in the corresponding locus, with 2 copies having a mutant allele.


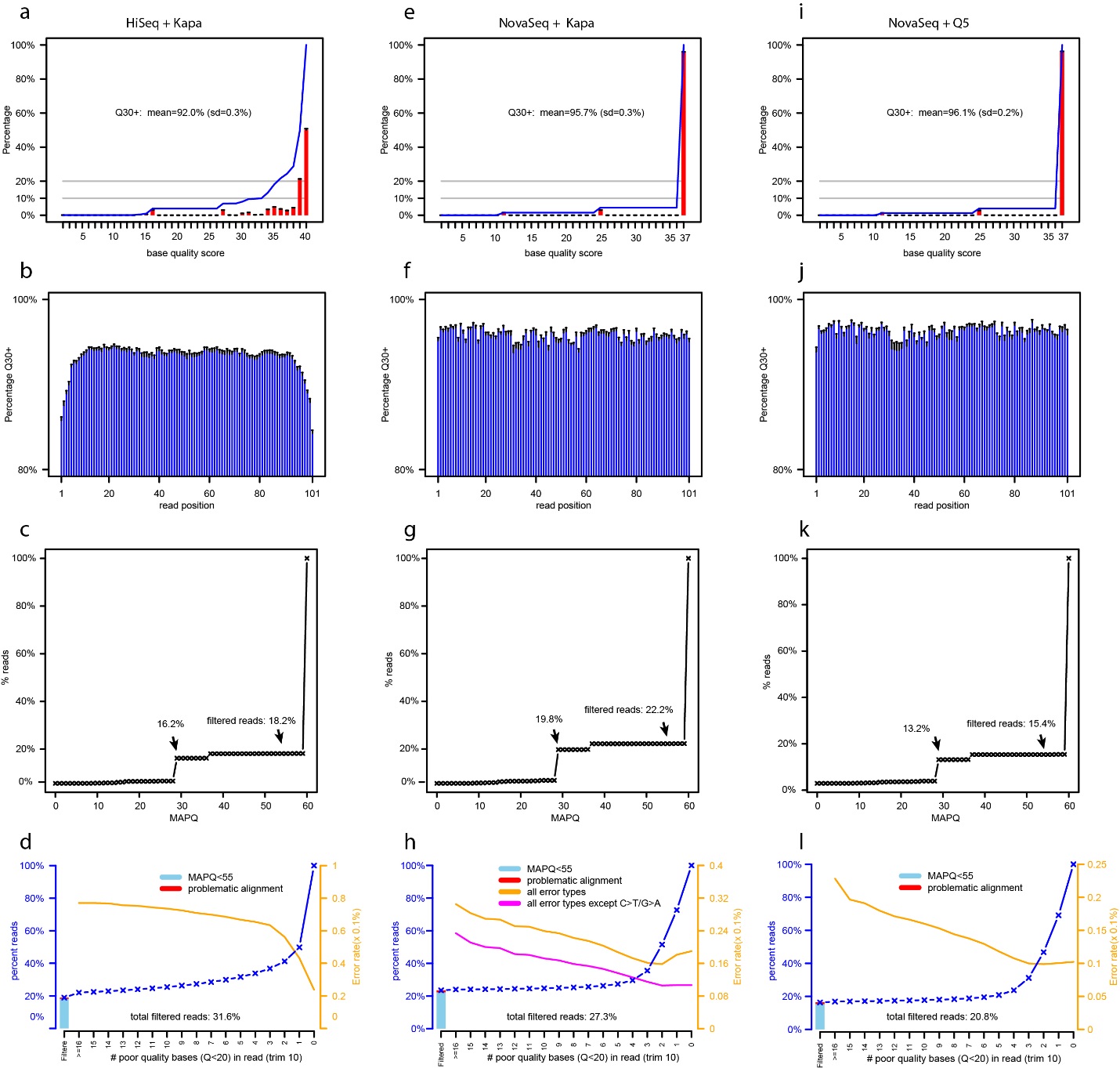


Figure S3. Quality metrics of sequenced datasets.

Base-pair quality score distribution (a), read position effect (b), mapping-quality distribution (c), and read-quality distribution (d) of HiSeq (left panels), NovaSeq with Kapa enzyme (middle panels, e, f, g, h), and NovaSeq with Q5 enzyme (right panels, i, j, k, l). For base-pair quality distribution (panels a, e, i), the cumulative distribution is shown as a blue line. The average number of bases with Phred quality ≥30 is indicated. In panels b, f, and j, the average percentage of bases with Phred quality ≥30 per read position is indicated. Error bars indicate standard deviation. In panels c, g, and k, the cumulative distribution of the MAPQ score is depicted (off-target captures were also counted), and the percentage of reads to be filtered is indicated as black arrows for cutoff values of 30 and 55, respectively. In panels d, h, and l, the percentage of reads with low MAPQ (<55) and potentially problematic aligment are indicated by the leftmost bar, followed by the percentage of reads with a given number of poor-quality bases (base quality score<20). Error rates in each quality bin are indicated with an orange line. The total percentage of reads filtered is indicated in each panel. For NovaSeq with Kapa enzyme (panel h), the read level error rate did not monotonically decrease with read quality (orange line). This observation is likely caused by C>T/G>A errors because removing them resulted in monotonic decrease (purple line).


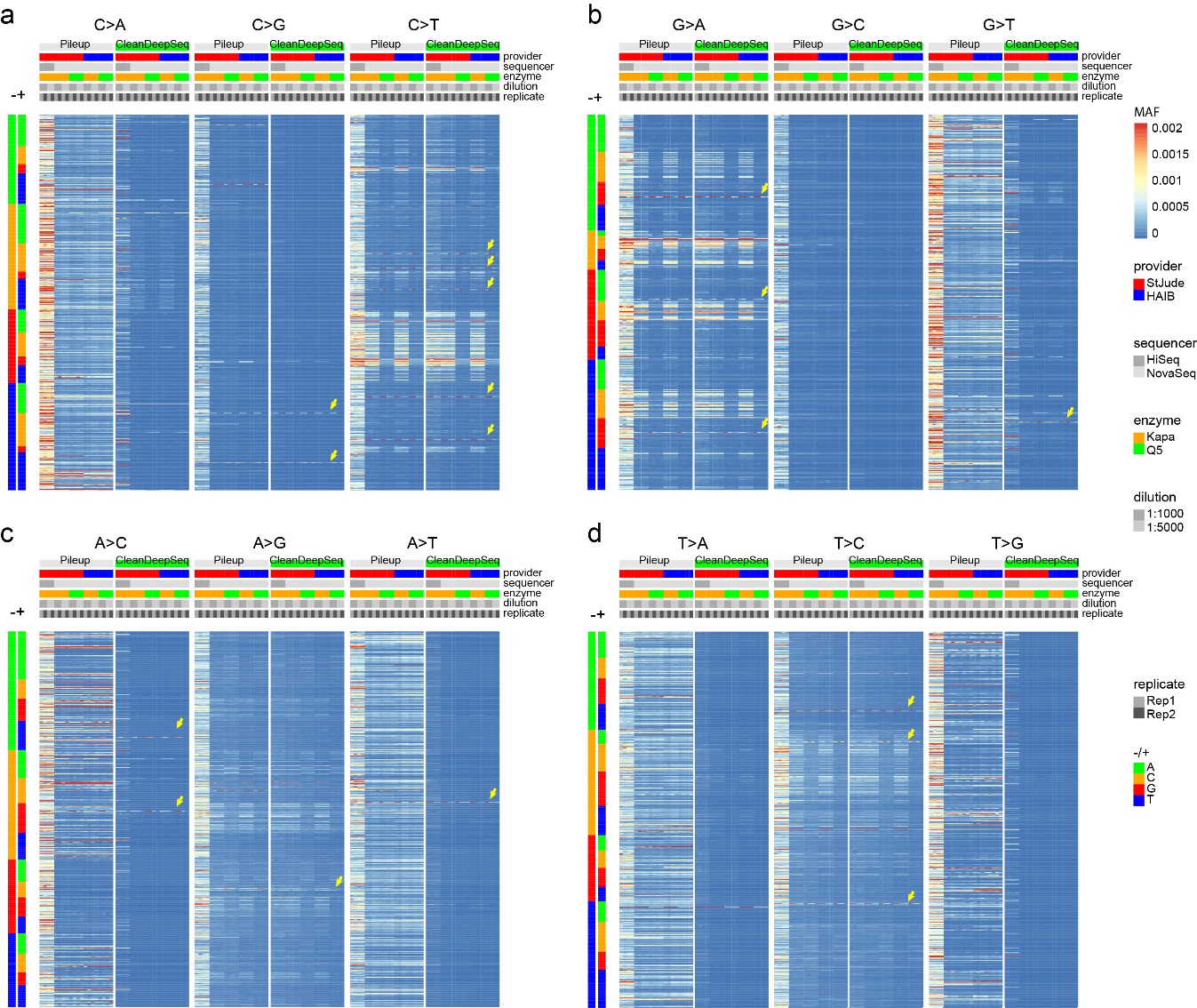


Figure S4. Heatmap of error profiles across sequencing providers, sequencers, PCR enzymes, replicates, and dilutions.

The genomic sites were categorized by genotypes C (a), G (b), A (c), and T (d) as heatmaps, where rows indicate genomic sites and columns indicate samples under diferrent experimental conditions indicated on top, including error rate calculation method (Pileup or CleanDeepSeq), sequencing provider (Genomics Laboratory, Department of Computational Biology, St. Jude Children’s Research Hospital [St. Jude] or Genomics Service Lab, HudsonAlpha Institute of Biotechnology [HAIB]), sequencer (HiSeq or NovaSeq), PCR enzyme (Kapa or Q5), dilution concentration (1:1000 or 1:5000), and replicate (Rep1 or Rep2). Within each genotype category, all three possible changes are depicted. Known somatic mutations are indicated by yellow arrows (Methods).


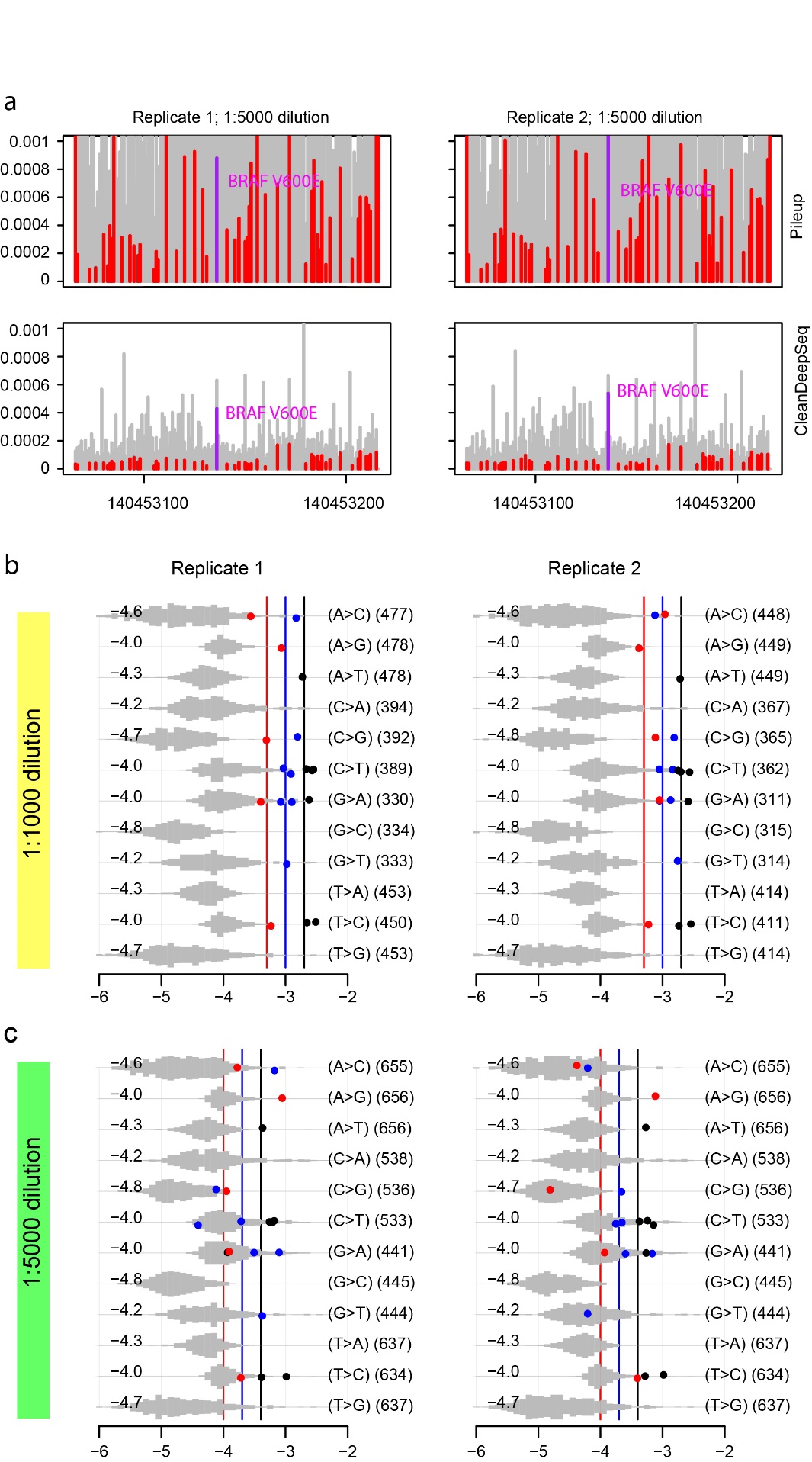


Figure S5. HiSeq error profile under CleanDeepSeq. See Fig. 2 for legends.


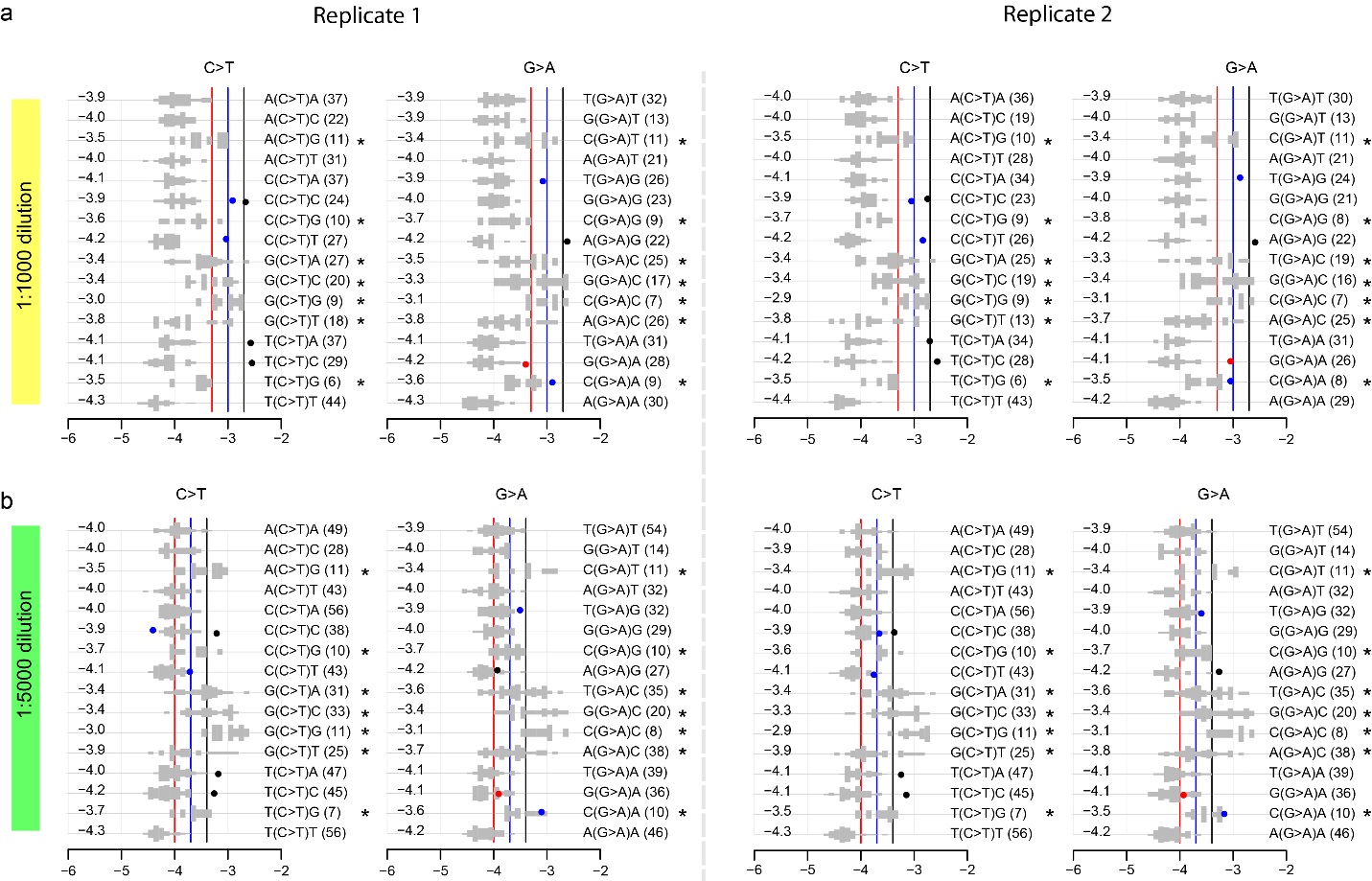


Figure S6. Context dependency of C>T/G>A errors in HiSeq data under CleanDeepSeq.

Contexts with an elevated error rate are highlighted with an asterisk.


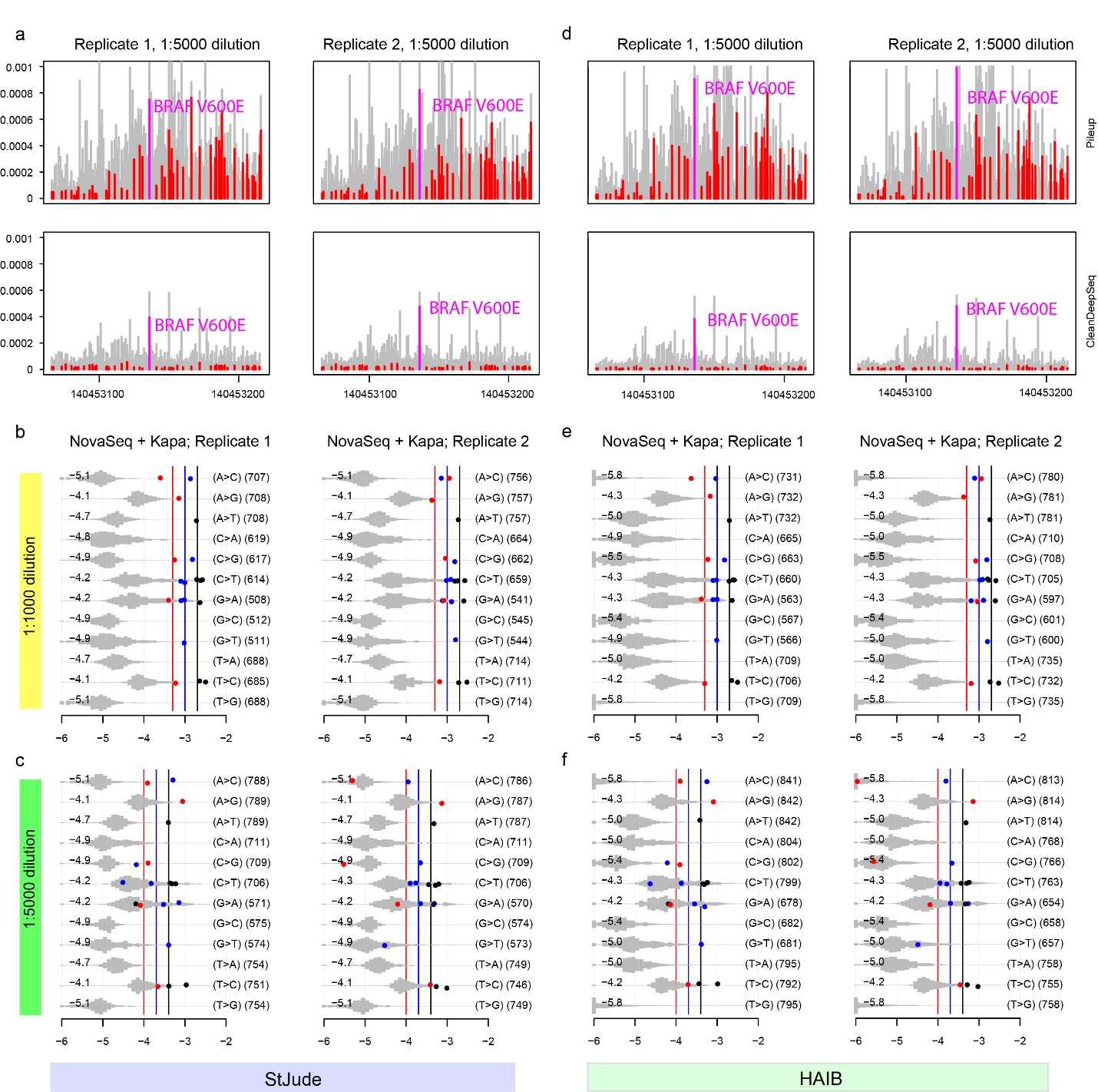


Figure S7. NovaSeq+Kapa error profile under CleanDeepSeq. See Fig. 2 for legends.

The StJude dataset (panels a, b, c) and HAIB dataset (panelc d, e, f) are included.


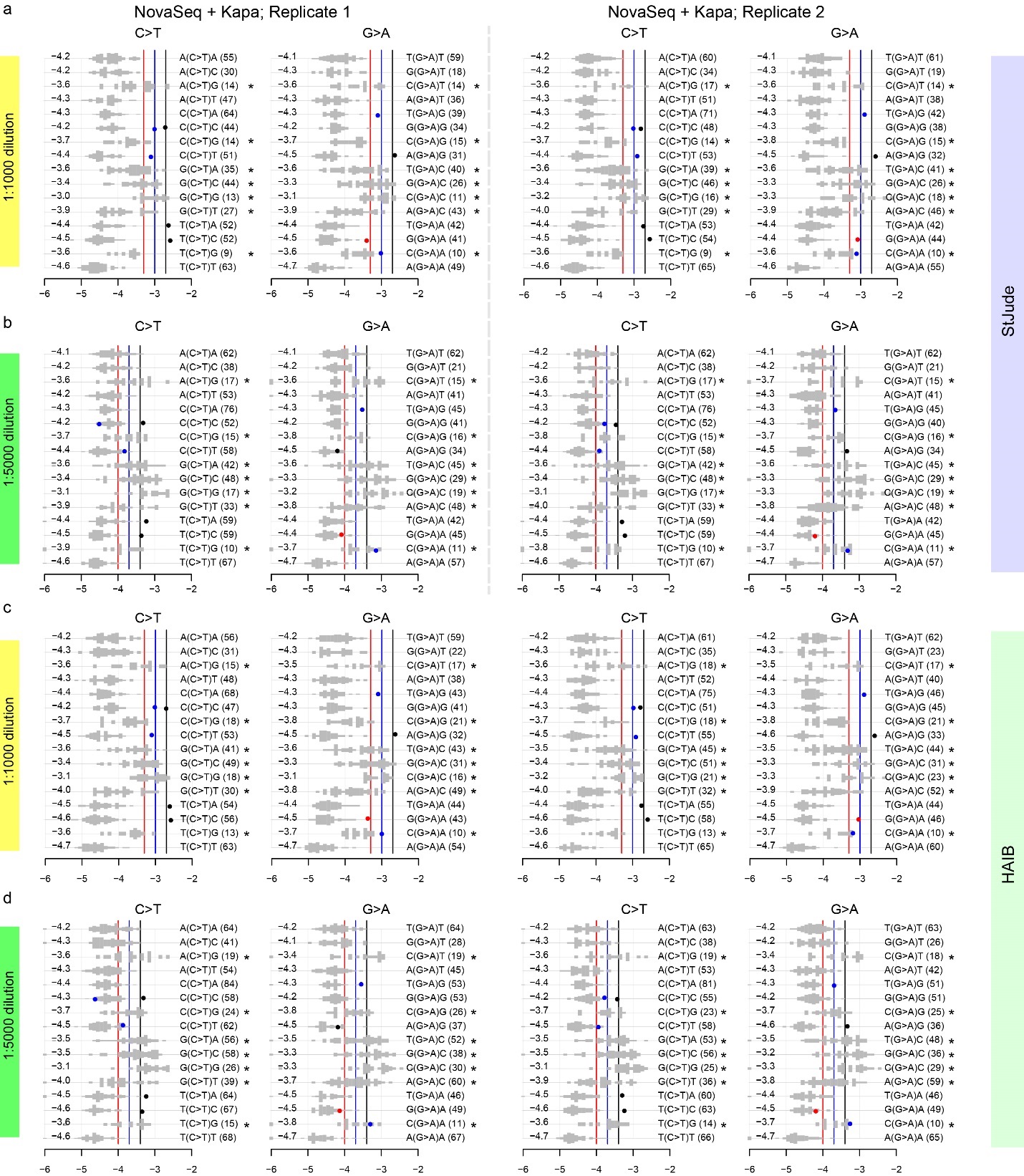


Figure S8. Context dependency of C>T/G>A errors in NovaSeq+Kapa dataset under CleanDeepSeq. The StJude dataset (panels a, b) and HAIB dataset (panels c, d) are included. Contexts with an elevated error rate are highlighted with an asterisk.


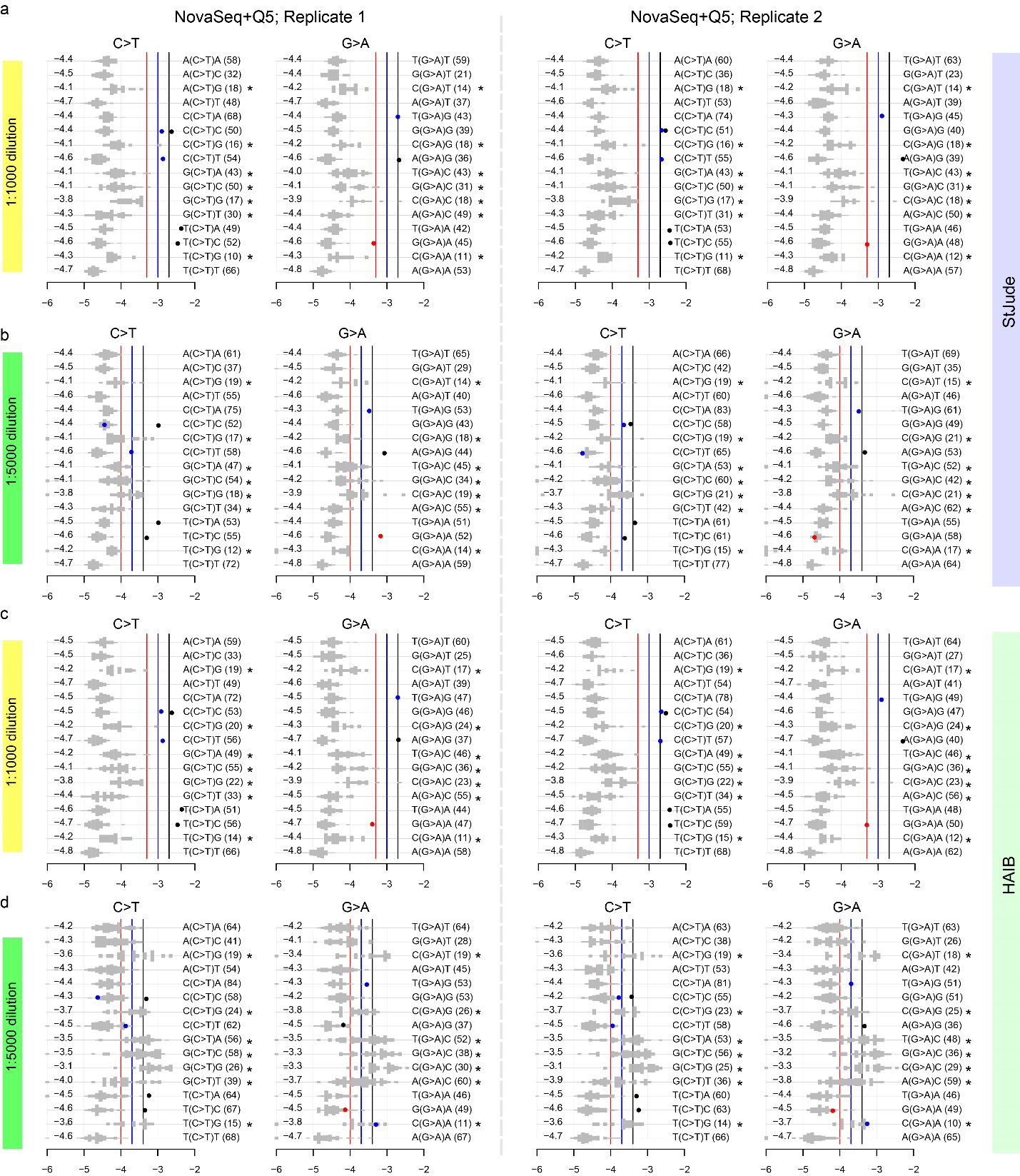


Figure S9. Context dependency of C>T/G>A errors in NovaSeq+Q5 dataset under CleanDeepSeq. The StJude dataset (panels a,b) and HAIB dataset (panels c,d) are included. Contexts with an elevated error rate are highlighted with an asterisk.


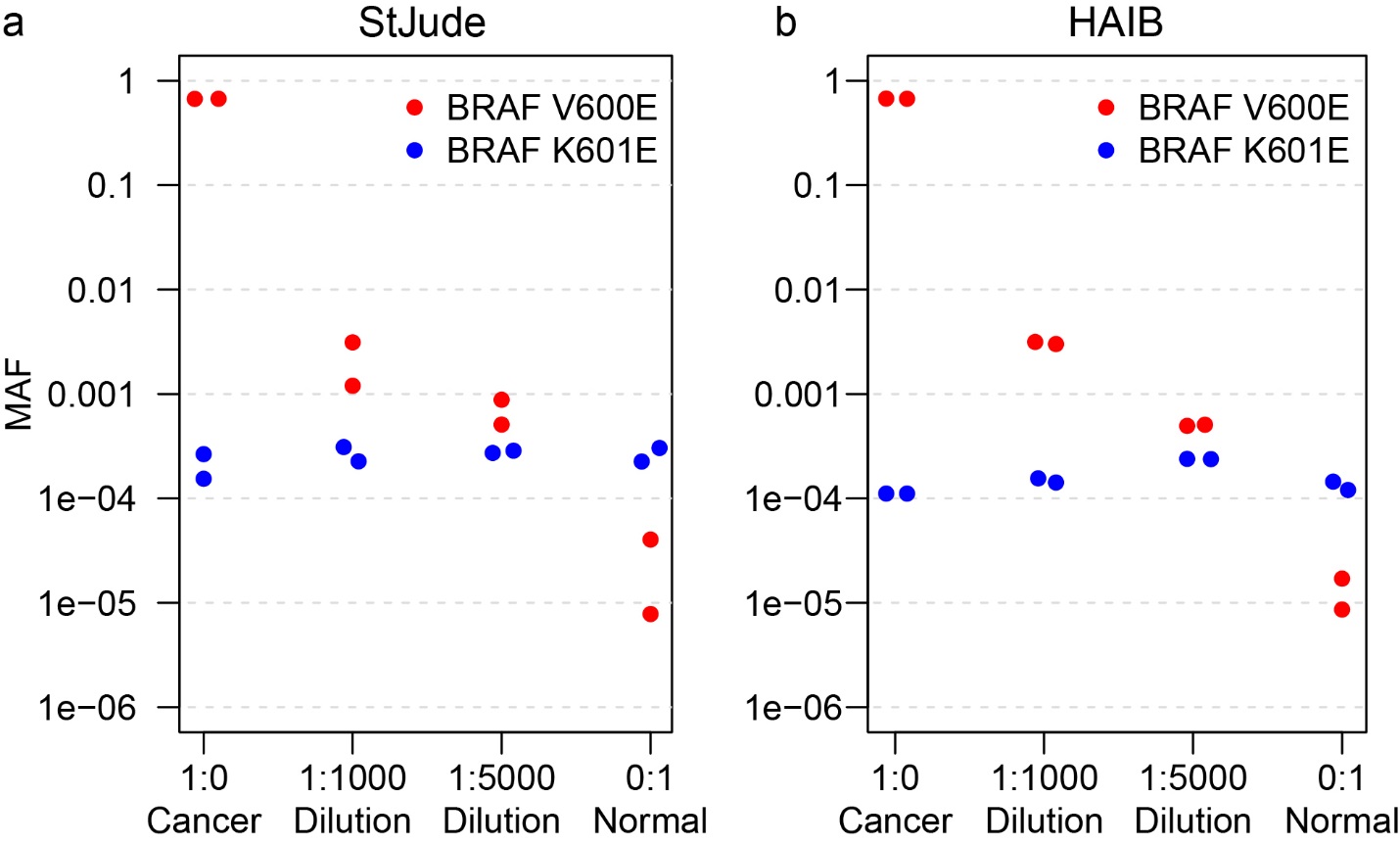


Figure S10. False-postive introduced by “forced calling”.

*BRAF* V600E (red dots) is a known somatic mutation in COLO829 and has a high MAF of 0.67 in undiluted cancer cell line (Cancer) in the two replicates of the StJude (a) and HAIB NovaSeq + Q5 datasets (b). Its MAF decreases with dilution ratio of 1:1,000 and 1:5,000, and its MAF is minimal in undiluted Normal sample, the latter reflecting background error rate. However, *BRAF* K601 is known to be wildtype in COLO829, which is also supported by its minimal MAF in the undiluted cancer cell line. It would be tempting to call K601E, a hotspot mutation, as “somatic” if the allele fractions of only 1:1,000 and 1:5,000 samples were examined. Clearly, this method would result in false-positive calls because K601E is a T>C change, which has an elevated error rate (Fig. 4b,c,e,f). Such false-postives could be easily avoided byusing our error profile analysis method.


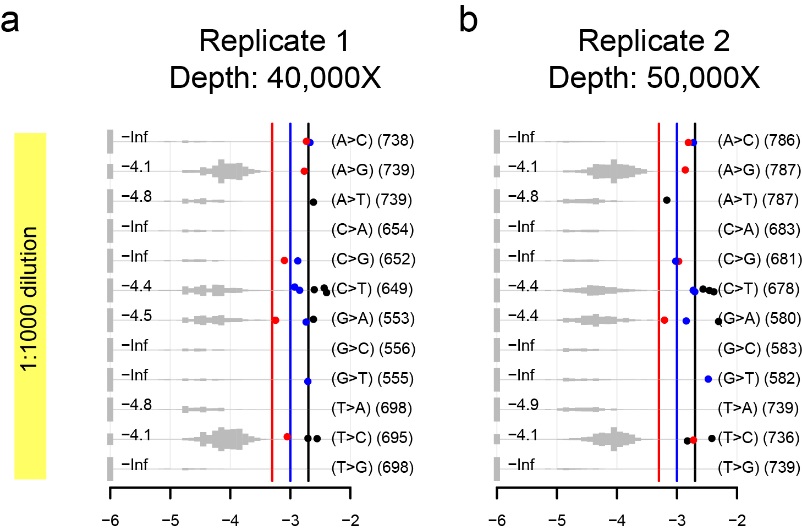


Figure S11. Error profiles in down-sampling of NovaSeq + Q5 dataset.

We performed down-sampling on the NovaSeq+Q5 1:1000 dilution dataset generated by St. Jude to obtain a median sequencing depth between ~40,000X to 50,000X. Note that the median background error rate (in log10 scale) is non-informative (indicated by “-Inf”) for low error rate substitutions because most sites have no mutant alleles.


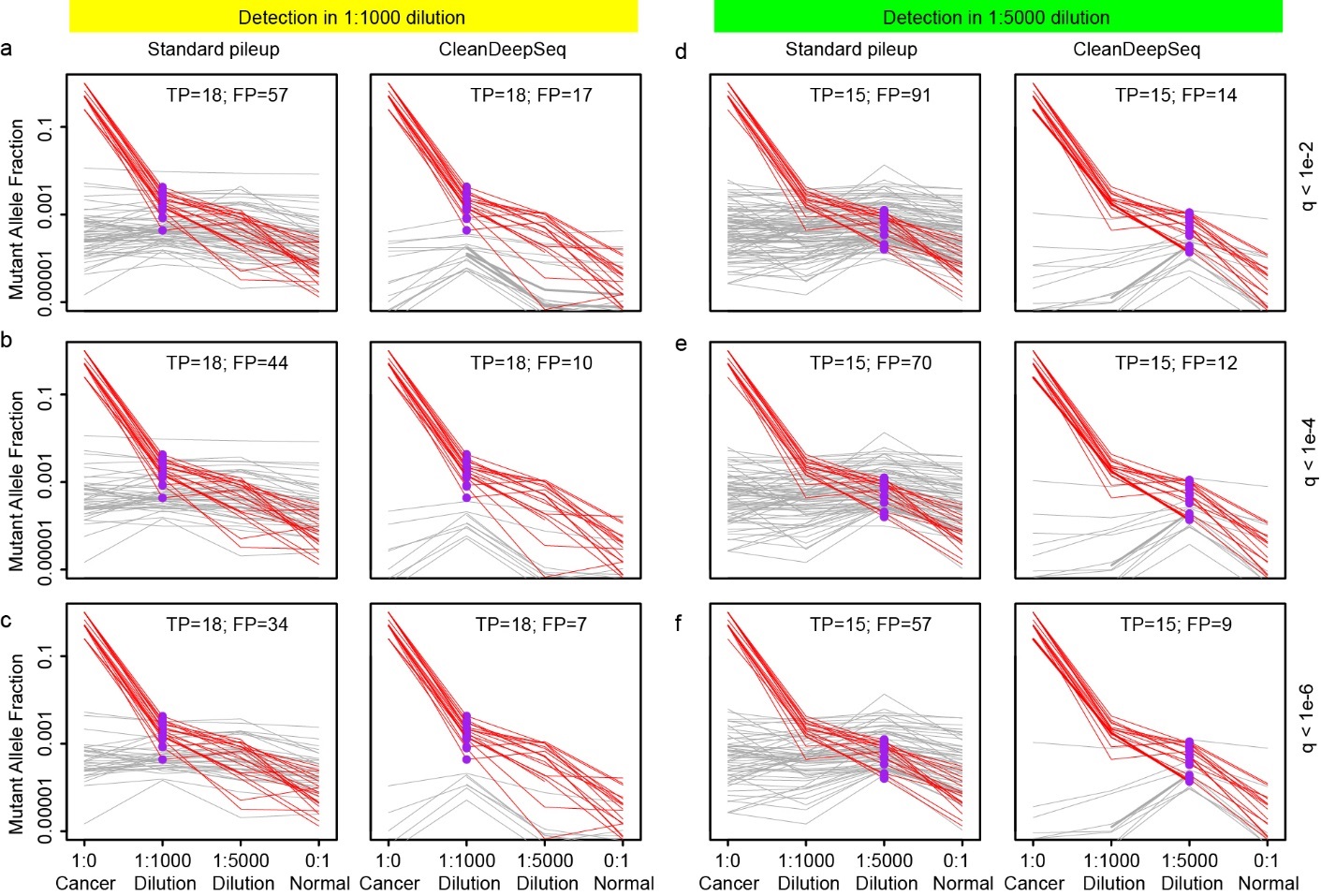


Figure S12. Comparison of standard pileup and CleanDeepSeq by using deepSNV on dilution experiments (NovaSeq + Q5 in StJude dataset) including 1:1,000 dilution (panels **a**,**b**,**c**) and 1:5,000 dilution (panels **d**,**e**,**f**) across three statistical significance cutoffs (q<10^-2^: panels **a**,**d**; q<10^-4^: panels **b**,**e**; and q<10^-6^: panels **c**,**f**, where q is the P value after Bonferroni correction). In each panel, mutant allele fraction (y-axis) of detected variants across undiluted cancer (1:0), 1:1,000 dilution, 1:5,000 dilution, and undiluted normal cells (0:1) are depicted to illustrate the false-positive (gray lines) and true-positive (red lines) status. Allele fractions in the index sample are colored purple. The total number of true-positives (TP) and false-positives (FP) are indicated. CleanDeepSeq led to 3- to 6-fold fewer false-positives than did standard pileup, without compromising sensitivity.


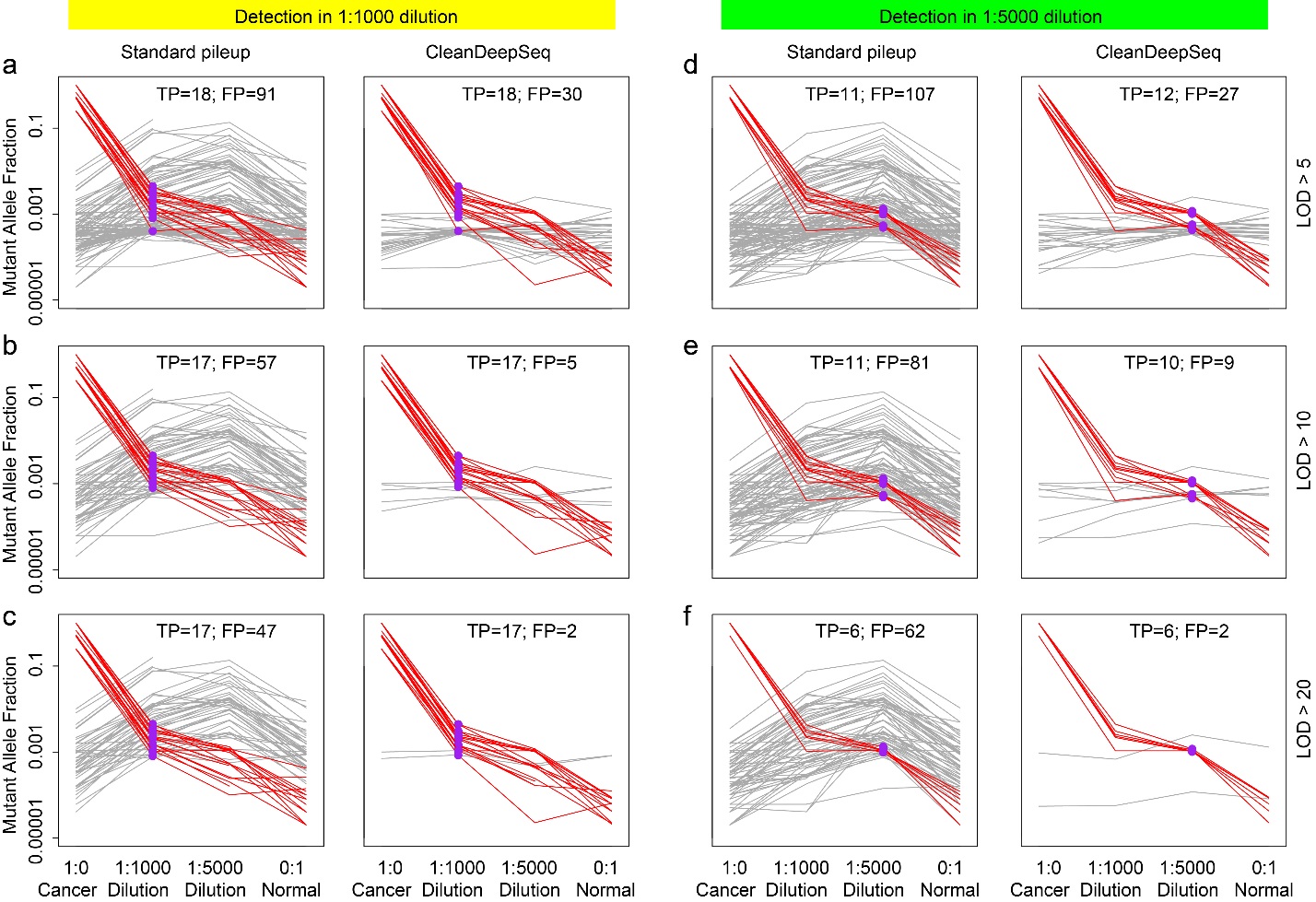


Figure S13. Comparison of standard pileup and CleanDeepSeq by using MuTect on dilution experiments (NovaSeq + Q5 in StJude dataset) including 1:1,000 (panels **a**,**b**,**c**) and 1:5,000 dilution (panels **d**,**e**,**f**) across three LOD cutoffs (LOD>5: panels **a**,**d**; LOD>10: panels **b**,**e**; LOD>20: panels **c**,**f**). In each panel, the mutant allele fraction (y-axis) of detected variants across undiluted cancer (1:0), 1:1,000 diltion, 1:5,000 dilution, and undiluted normal cells (0:1) is depicted to illustrate the false-positive (gray) and true-positive (red) status. Allele fractions in the index sample are colored purple. The total number of true-positives (TP) and false-positives (FP) is indicated. Compared with standard pileup, CleanDeepSeq leads to 3- to 30-fold reduction of false-positives, without compromising sensitivity.
